# A Highly Ordered Nitroxide Side Chain for Distance Mapping and Monitoring Slow Structural Fluctuations in Proteins

**DOI:** 10.1007/s00723-023-01618-8

**Published:** 2023-10-14

**Authors:** Mengzhen Chen, Tamás Kálai, Duilio Cascio, Michael D. Bridges, Julian P. Whitelegge, Matthias Elgeti, Wayne L. Hubbell

**Affiliations:** 1 Jules Stein Eye Institute and Department of Chemistry and Biochemistry, University of California, Los Angeles, CA 90095 USA; 2 Institute of Organic and Medicinal Chemistry, Faculty of Pharmacy, University of Pécs, Szigeti St. 12, Pecs, 7624 Hungary; 3 Department of Biological Chemistry, UCLA-DOE Institute, Howard Hughes Medical Institute, and Molecular Biology Institute, University of California, Los Angeles, CA 90095 USA; 4 The Pasarow Mass Spectrometry Laboratory, David Geffen School of Medicine, The Jane and Terry Semel Institute for Neuroscience and Human Behavior, University of California, Los Angeles, CA 90095 USA; 5 Present Address: Institute for Drug Discovery, Leipzig University Medical Center, Härtelstr. 16-18, 04107 Leipzig, Germany

## Abstract

Site-directed spin labeling electron paramagnetic resonance (SDSL-EPR) is an established tool for exploring protein structure and dynamics. Although nitroxide side chains attached to a single cysteine via a disulfide linkage are commonly employed in SDSL-EPR, their internal flexibility complicates applications to monitor slow internal motions in proteins and to structure determination by distance mapping. Moreover, the labile disulfide linkage prohibits the use of reducing agents often needed for protein stability. To enable the application of SDSL-EPR to the measurement of slow internal dynamics, new spin labels with hindered internal motion are desired. Here, we introduce a highly ordered nitroxide side chain, designated R9, attached at a single cysteine residue via a non-reducible thioether linkage. The reaction to introduce R9 is highly selective for solvent-exposed cysteine residues. Structures of R9 at two helical sites in T4 Lysozyme were determined by X-ray crystallography and the mobility in helical sequences was characterized by EPR spectral lineshape analysis, Saturation Transfer EPR, and Saturation Recovery EPR. In addition, interspin distance measurements between pairs of R9 residues are reported. Collectively, all data indicate that R9 will be useful for monitoring slow internal structural fluctuations, and applications to distance mapping via dipolar spectroscopy and relaxation enhancement methods are anticipated.

**Supplementary Information:**

The online version contains supplementary material available at 10.1007/s00723-023-01618-8.

## Introduction

**Fig. 1:**
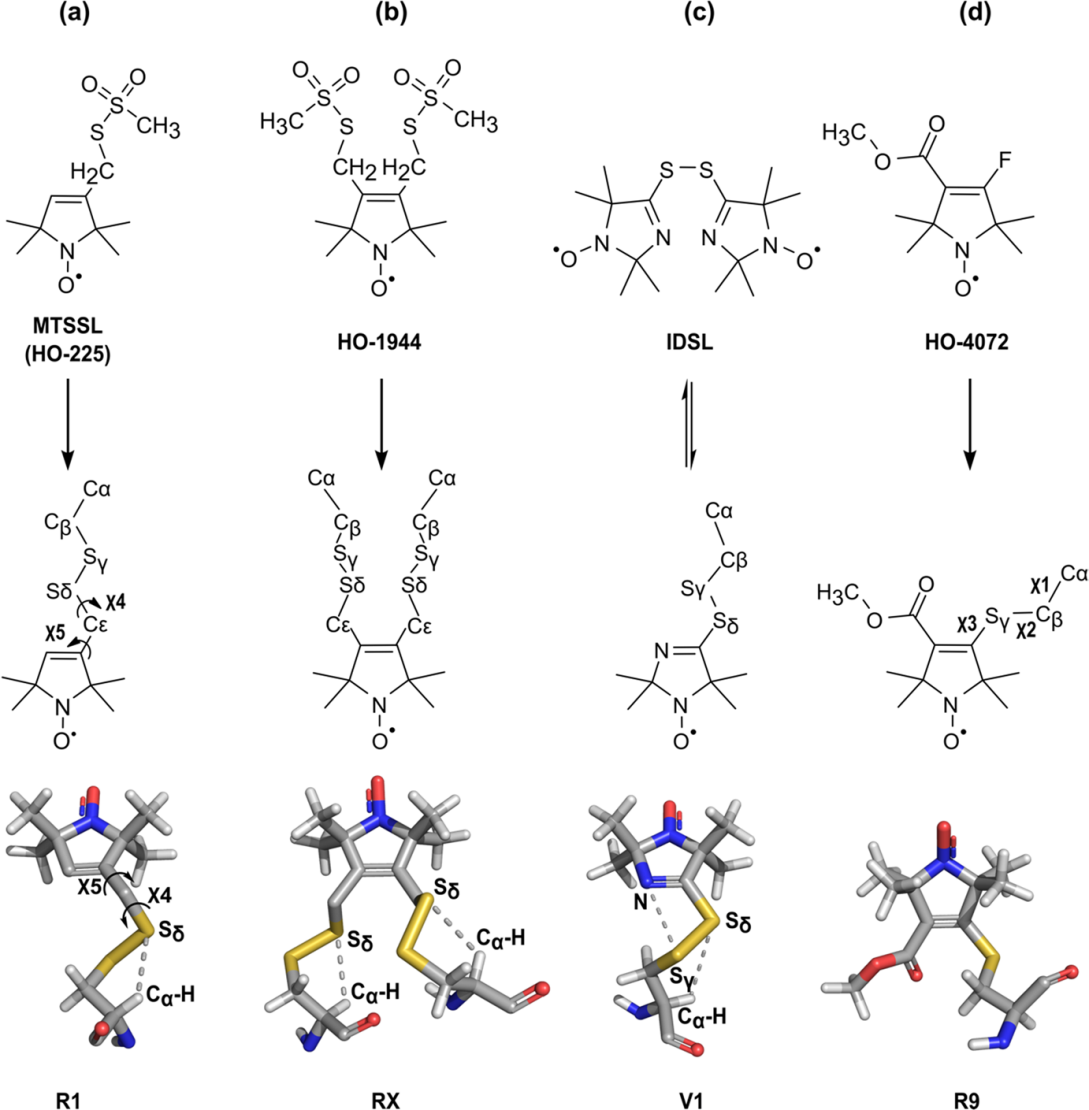
Structures of spin labeling reagents and reaction products with cysteine residues on proteins involved in this study, among which the reagent HO-4072/the side chain R9 is the focus. **a** Structure of the reagent 1-oxyl-2,2,5,5-tetramethypyrroline-3-methyl (MTSSL, HO-225), the R1 side chain with designations of atoms and dihedral angles used in the text, and the molecular model (from PDB code 2CUU). The C_α_-H···S_δ_ interaction that restricts the motion of the disulfide linkage is shown. **b** Structure of the reagent 3,4-bis-(methanethiosulfonylmethyl)-2,2,5,5-tetramethyl-2,5-dihydro-1H-pyrrol-1-yloxy radical (HO-1944), the RX side chain, and the molecular model (from PDB code 5LWO). **c** Structure of the reagent bis (2,2,5,5-tetramethyl-3-imidazoline-1-oxyl-4-il)-disulfide (IDSL), the V1 side chain with designations of atoms and dihedral angles used in the text, and the molecular model (from PDB code 3K2R). The C_α_-H···S_δ_ interaction and the N···S_γ_ interaction are shown. **d** Structure of the reagent methyl 4-fluoro-2,2,5,5-tetramethyl-2,5-dihydro-1H-pyrrole-3-carboxylate-1-yloxyl radical (HO-4072), the R9 side chain with designations of atoms and dihedral angles used in the text, and the molecular model (from PDB code 8TAT, see Result section of this article)

Methanethiosulfonate nitroxides are extensively used as reagents for spin labeling cysteines in proteins due to their high reactivity and specificity [1–3]. The most popular reagent (MTSSL) generates a disulfide-linked side chain designated R1 (Fig. 1a). The high reactivity of the methanethiosulfonate functionality allows facile derivatization of partially and even fully buried cysteine residues [3, 4]. The conformation and internal dynamics of R1 in proteins have been extensively characterized by crystallography [5–9], EPR spectral simulations [4, 10, 11], and density functional theory computations [12]. An important feature of R1 that has emerged from these studies is the interaction of the S_δ_ sulfur of the disulfide linkage with the C_α_–H hydrogen of the residue in helical structures (Fig. 1a). This interaction constrains the conformational space and gives rise to an anisotropic motion on the ns time scale described by torsional oscillations about the *χ*_4_/*χ*_5_ dihedral angles [4, 8]. The anisotropic motion averages the magnetic tensor elements of the nitroxide in such a manner as to make the continuous-wave electron paramagnetic resonance (CW-EPR) spectral lineshape exquisitely sensitive to fast (ns) structural fluctuations, and to modulation of the side chain motion by local interactions. Thus, R1 has become a sensor of choice for monitoring fast backbone fluctuations and mapping secondary and tertiary structures of the protein [11, 13–15].

However, the internal motion of R1 limits its utility for monitoring slow protein internal motions (μs–ms), for example, using saturation transfer electron paramagnetic resonance (ST-EPR [16–18]) or pulsed electron–electron double resonance (ELDOR [19]) methods. Moreover, the existence of multiple rotamers [8] in addition to the internal motion leads to uncertainty in the location of the nitroxide and hence in the interpretation of interspin distance distributions determined from pulsed dipolar spectroscopy [20, 21].

To limit the conformational space and restrain the motion of the nitroxide, we have introduced two other disulfide-linked side chains. The first, designated RX [22], crosslinks pairs of suitably placed cysteine residues in α-helices and β-sheets (Fig. 1b) using a bi-functional methanethiosulfonate reagent (HO-1944, Fig. 1b). Due to constraints imposed by the two sites of attachment, the RX conformational space is highly constrained and the limited internal motion is strongly ordered [22–24]. Another, designated V1 [25], is introduced at a single cysteine using a disulfide-linked imidazoline nitroxide biradical (IDSL, Fig. 1c) via a thiol/disulfide exchange reaction [26, 27]. V1 has a highly restricted internal motion resulting from the same C_α_-H···S_δ_ interaction as in R1, but in addition a unique N···S_γ_ intra-side chain interaction [25, 28] (Fig. 1c). The strong ordering and localization make RX and V1 attractive for use in distance measurements [22, 28] and for slow motion detection [22, 29–31].

Despite the positive features of these disulfide-linked spin label side chains, there are some disadvantages. First, the disulfide is readily cleaved by reducing agents that are necessary for the stability and activity of some proteins. In the case of V1, the reactive disulfide is relatively unstable and susceptible to spontaneous hydrolytic cleavage in aqueous solution. Moreover, the high reactivity of methanethiosulfonate and IDSL reagents to even buried cysteine residues requires the removal of at least some native cysteines [3, 25]. RX necessitates the introduction of two cysteine residues, and the cross-link can stabilize strained, non-native structures.

In this study, we introduce a new nitroxide reagent (HO-4072) that reacts exclusively with solvent-exposed cysteine residues (Fig. 1d). The nitroxide side chain formed by the reaction, designated R9, has a stable thioether linkage that tolerates the commonly used reducing reagents and has a short linker that positions the nitroxide close to the protein backbone. Crystal structures are presented that identify unique interactions that lead to a generally high ordering of R9.

## Results

The R9 side chain has been introduced at solvent-exposed sites in T4 Lysozyme (T4L) carrying cysteine mutations in the pseudo wild-type background [32–34]. In the sections below, we characterize the reaction mechanism and rate of HO-4072 with cysteine residues, explore the internal dynamics of the R9 side chain using various EPR methods, and present X-ray crystal structures of R9 on helical sites and identify the structural origin of the strongly ordered motion. Applications to determine inter-residue distance with double electron–electron resonance (DEER), and to monitor slow protein dynamics with ST-EPR are presented. Spin–lattice relaxation times in T4L are measured via Saturation Recovery (SR-EPR [29, 35, 36]) to evaluate the potential of R9 for measuring distances via relaxation enhancement.

### Reaction Mechanism and Kinetics

**Fig. 2:**
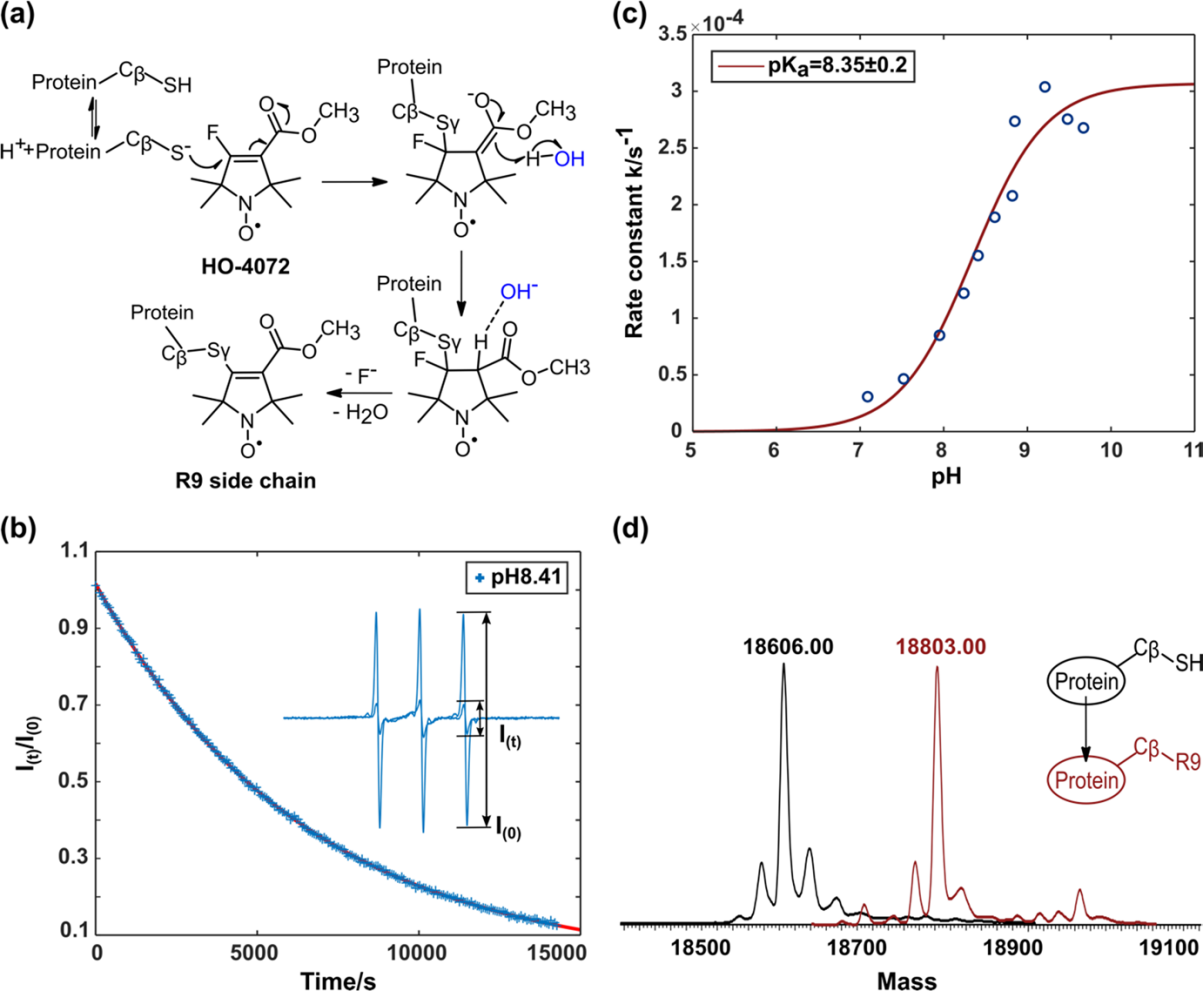
Characterization of the reaction of HO-4072 with cysteine. **a** Proposed spin-label reaction mechanism with HO-4072 to generate the R9 side chain. **b** The disappearance of the CW-EPR signal (*I*_(t)_/*I*_(0)_, see Materials and Methods) for unreacted HO-4072 as a function of time in the presence of a limiting amount of HO-4072. This example is recorded at pH 8.41 in solution. Under these conditions, the reaction is pseudo-first order. The trace (red) fit to the data points (blue) is a monoexponential with rate constant $k^{\prime}_{1}$  = 1.552 (± 0.01) × 10^–4^ s^−1^. **c** Plot of the reaction rate constant, determined as for (**b**), vs pH. The fit to the data is a Henderson–Hasselbalch equation and yields a p*K*_a_ for cysteine of 8.35 ± 0.2. **d** Deconvoluted ESI mass spectra before and after the spin-label reaction between HO-4072 and T4L mutant with a single solvent-exposed cysteine

The reaction of HO-4072 with cysteine is anticipated to proceed via a Michael addition followed by E1 elimination (Fig. 2a). The Michael addition should proceed most rapidly in the alkaline solution with RS^−^ as the nucleophile. Reaction progress is readily followed by the decrease in sharp spectral component corresponding to unreacted HO-4072 as it reacts under pseudo-first-order conditions with a large excess of protein (Fig. 2b; see Materials and Methods). Figure 2c shows the pseudo-first order rate constant determined in this manner as a function of pH for T4L 72C. The apparent p*K* determined by fitting is 8.35 ± 0.2, a reasonable value for the p*K*_a_ of cysteine in a protein [37].

However, these data do not identify reaction product(s), and it could in principle be the intermediate preceding the elimination of the HF molecule (Fig. 2a). Moreover, the 4-ester could be partially or fully hydrolyzed at pH ≥ 8 in which the reaction is favorably carried out. Thus, a mixture of products is possible. However, mass spectrometry indicates a major species with a mass increase of 196 ± 1 Da corresponding to the addition of the R9 side chain (Fig. 2d), which is readily distinguishable from the intermediate (a theoretical 216 ± 1 Da increase). Moreover, the mass spectra recorded at different times during the reaction did not reveal any significant accumulation of reaction intermediates (Fig. S1), indicating that the final elimination step is rapid. In a separate experiment, it was found that ester hydrolysis of a thioether analog of R9 does not take place at a measurable rate up to a pH 8.7, *T* = 28 ℃, within 48 h (see details in SI Materials and Methods, Fig. S2).

The cysteine-less T4L was also incubated with the HO-4072 under the same reaction conditions. It was determined that background labeling of groups other than cysteine contributed less than 1% of the total EPR signal of the labeled cysteine mutants.

### Internal Motion of R9 on a Solvent Exposed Helix Site

X-band CW-EPR spectral lineshapes of spin-labeled proteins encode information of the nitroxide dynamics on the ps-ns time scale [15]. The rotational diffusion of the entire protein, backbone fluctuations, and internal side chain motions that can be further modulated by local interactions [3, 10, 11] occur in this time range.

To isolate the side chain and protein dynamic modes, the spin-labeled protein is either investigated in a high viscosity medium or immobilized on a solid support to reduce or eliminate rotational diffusion of the entire protein [3, 38, 39]. Further, contributions from backbone fluctuations and local interactions can be minimized by selecting a reference site for labeling in rigid sequences where the side chain is solvent-exposed and non-interacting with other residues. Site 72 in T4L meets these criteria as it is located in the center of the long and rigid Helix C [3, 4].

**Fig. 3:**
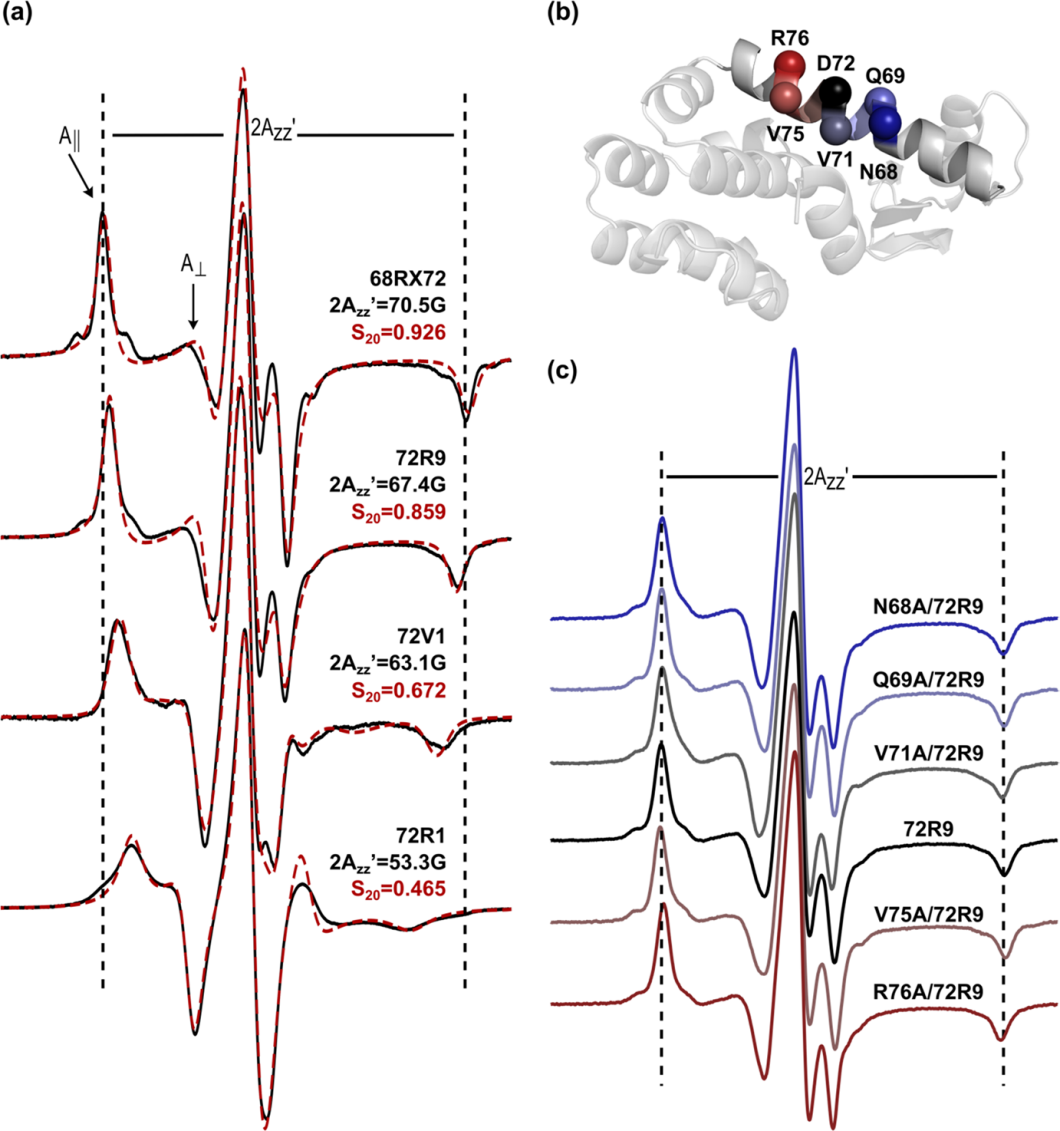
X-band CW-EPR spectra and simulations for spin labels on T4L attached to Sepharose solid supports. **a** Experimental spectra of T4L 68RX72, 72R9, 72V1, and 72R1 at room temperature (black solid traces) and the corresponding simulated spectra (red dashed traces). The parallel (A_∥_) and perpendicular (A_⊥_) components of the axially symmetric hyperfine tensor, and the effective hyperfine splitting, $2A^{\prime}_{zz}$ are shown. The vertical lines are drawn and fixed to the value corresponding to the $2A^{\prime}_{zz}$ of the T4L 68RX72 spectrum for comparison. The corresponding values of the *S*_20_ are provided. **b** Ribbon diagram of T4L (PDB code 1L63) showing the sites where R9 is introduced and neighboring residues which are replaced with alanine (indicated by the respective C_α_ atom). The C_α_ atoms are colored corresponding to the spectra in **c** below. **c** Corresponding CW-EPR spectra of T4L 72R9 with background mutations indicated in **b** show the effect of alanine-replacement of neighboring side chains on R9 side chain dynamics. The vertical lines are drawn and fixed to the value corresponding to the $2A^{\prime}_{zz}$ of the 72R9 spectrum for comparison. The magnetic field scan width for spectra in **a** and **c** is 100 G

Thus, the CW-EPR spectra of the side chains shown in Fig. 1 at site 72 should reveal the relative side chain internal motions on the ps-ns time scale, in the absence of backbone contributions. Figure 3a (black solid traces) shows the CW-EPR spectra for RX cross-linked between sites 68 and 72 (68RX72), and for 72R9, 72V1, and 72R1; in each case, the protein is immobilized on solid supports (CNBr-Sepharose [38, 39]). The spectrum of 68RX72 is striking with well-resolved parallel (A_∥_) and perpendicular (A_⊥_) components of the hyperfine tensor, narrow linewidths for the outer hyperfine extrema, and a large effective hyperfine splitting $2A^{\prime}_{zz}$ [40]. In agreement with published results, the spectrum is well fit by a Microscopic Order Macroscopic Disorder model (MOMD [41]) (red dashed trace) corresponding to a highly ordered but rapid anisotropic motion [22].

In the current implementation of MOMD, the 2*p* orbital of the nitroxide is constrained to move within a cone and the amplitude of motion is measured by an order parameter $S_{20} = 1/2\langle (3\cos^{2} \alpha - 1)\rangle$, where α is the half angle of the cone. The values of *S*_20_ lie between 0 and 1 and increase with increasing order. The rate of motion is measured by an effective correlation time, *τ*_R_, values of which values increase with decreasing rate. For 68RX72, *S*_20_ = 0.93 and *τ*_R_ = 2.35 ns. The high order corresponding to *α* = 12° is due to both the backbone rigidity near site 72 and the highly constrained internal motion of the cross-linked RX side chain.

The spectra of 72R9, 72V1, and 72R1 each resemble that of 68RX72 but with decreasing $2A^{\prime}_{zz}$ and increasing linewidth. Remarkably, the lineshapes of each side chain can be simulated reasonably well by the same MOMD model and correlation time as RX but with varying degrees of order (Fig. 3a, red dashed traces). While small variations in other simulation parameters may further improve the fit quality (see Table S1, legend), it is apparent that spectral changes are dominated by the variation of the order parameter.

Within the context of this motional model, RX clearly has the most constrained (ordered) motion, but R9 with *S*_20_ = 0.86 (*α* = 18°) is similar and the most highly ordered among the singly attached spin label side chains. Note from Fig. 3a that the value of $2A^{\prime}_{zz}$ is a qualitative measure of the amplitude of motion, i.e., the order, and that there is also a systematic increase in spectral linewidths proceeding from RX to R1.

The short linkage of the R9 side chain positions the nitroxide close to the backbone, and it is possible that the high order of R9 may be the result of interactions with a neighboring residue. To test this, each nearest neighbor was mutated, one at a time, to alanine [3]. Figure 3b shows the locations of the five residues mutated (N68, Q69, V71, V75, and R76) and Fig. 3c provides the corresponding, essentially indistinguishable, EPR spectra. This finding shows that no single side chain interaction can account for the high degree of immobilization, but does not eliminate the unlikely possibility that multiple side chains act in a concerted fashion, while any single mutation has no effect. The methyl groups of alanine could interact with R9, a possibility that will be revisited below.

### Dependence of R9 Side Chain Mobility on the Local Structure

**Fig. 4:**
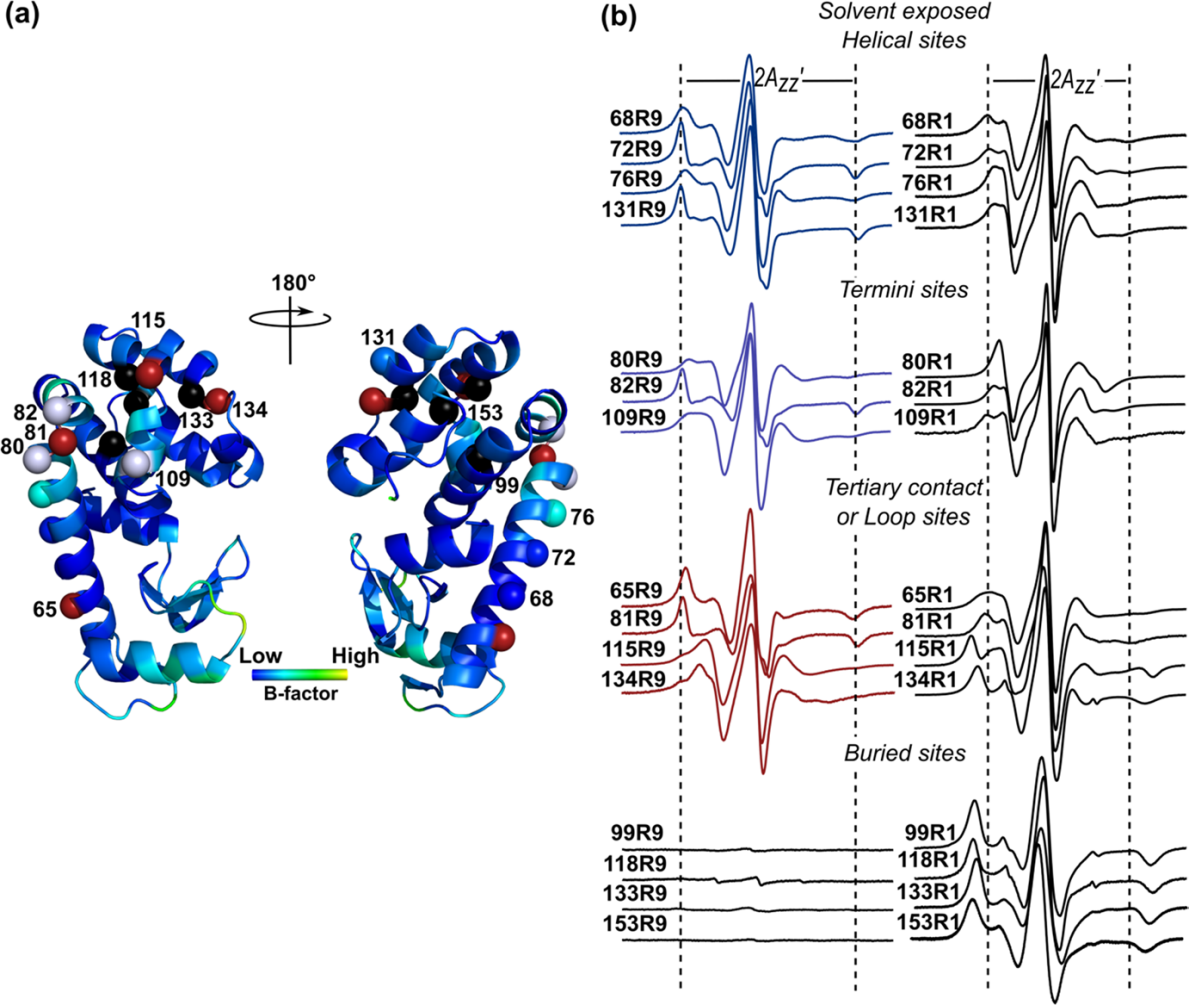
CW-EPR spectra of R9 and R1 at various sites of T4L. **a** Ribbon diagram of T4L (PDB code 1L63) showing all the sites used in this study, of which each residue is colored based on the value of B-factor. The blue spheres (non-interacting helical sites), blue–white spheres (helical termini sites), red spheres (tertiary contact or loop sites), and black spheres (buried sites) at the C_α_ indicate where spin labels are introduced. **b** CW-EPR spectra of R9 and R1 at room temperature in 30% (wt/wt) sucrose solution. The vertical lines are drawn and fixed to the value corresponding to the $2A^{\prime}_{zz}$ of the 72R9 or 72R1 spectrum as a reference for comparison. Spectra of buried sites are measured under the same experimental conditions to reveal how negligible the amount of a buried site can be labeled by R9

To explore the generality of labeling and the information content of R9 EPR spectra in a helical protein, the side chains were introduced, one at a time, at sites in distinct structural elements of T4L [3], namely solvent-exposed sites in the interior of regular helices (sites 68, 72, 76, 131), at the N-termini (82, 109) and a C-terminus (80), at an interhelical loop site (81), and at tertiary contact sites (65, 115, 134). No significant labeling was observed for cysteines at any of the buried sites tested (99, 118, 133, 153). Figure 4a shows the location of the above sites in T4L with the backbone color-coded according to crystallographic B-factors. Corresponding EPR spectra (Fig. 4b) of R9 and R1 at the same sites in T4L are shown for comparison; R1 spectra have all been previously published [3, 6, 25].

The EPR spectra of all labeled mutants were measured in a 30% (wt/wt) sucrose solution to minimize the rotational diffusion of the protein. Considering the variety of topographical regions involved, the solid-support attachment was not used to avoid potential interactions between nitroxide and the solid support which may differently contribute to the side chain dynamics [39].

First, the spectra reveal that the R9 side chain can be introduced with overall good yields at sites in any of the structural elements tested (Fig. 4b), except for the buried sites where there is essentially no labeling. On the other hand, the methanethiosulfonate R1 reagent (and disulfide V1 reagent) reacts readily with these sites and yields spectra reflecting strong immobilization of the side chain, as previously reported [3, 6, 25]. The selectivity for solvent-exposed sites is an important advantage of the R9 labeling scheme.

At all sites, the spectra of R9 reflect lower mobility compared to R1 as reflected in values of $2A^{\prime}_{zz}$. In particular, R9 side chains at sites in the center of ordered helices are strongly immobilized. However, in other regions, a site-specific variation in mobility is evident that may encode, among other things, information on local dynamics and interactions. Although an analysis of the structural and dynamical origin for the site-dependent mobility is beyond the scope of this work, crystal structures presented below identify the unique interactions of R9 that give rise to relative immobilization and offer clues to the origin of the site-dependent variation.

### Crystal Structure of T4L 65R9/76R9

To identify the interactions that constrain the internal motion of R9 at helical sites, a 1.60 Å crystal structure of T4L containing R9 side chains at positions 65 and 76 in Helix C (T4L 65R9/76R9) was solved and refined to an R-factor of 19.8%; refinement statistics are provided in SI (Table S2). A structure of R9 at reference site 72 could not be obtained, since this site is involved in crystal contact [42]. There is little difference between the backbone dihedral angles (Fig. S3) along Helix C in T4L 65R9/76R9 and the T4L structures, indicating that R9 does not distort the local helical geometry.

**Fig. 5:**
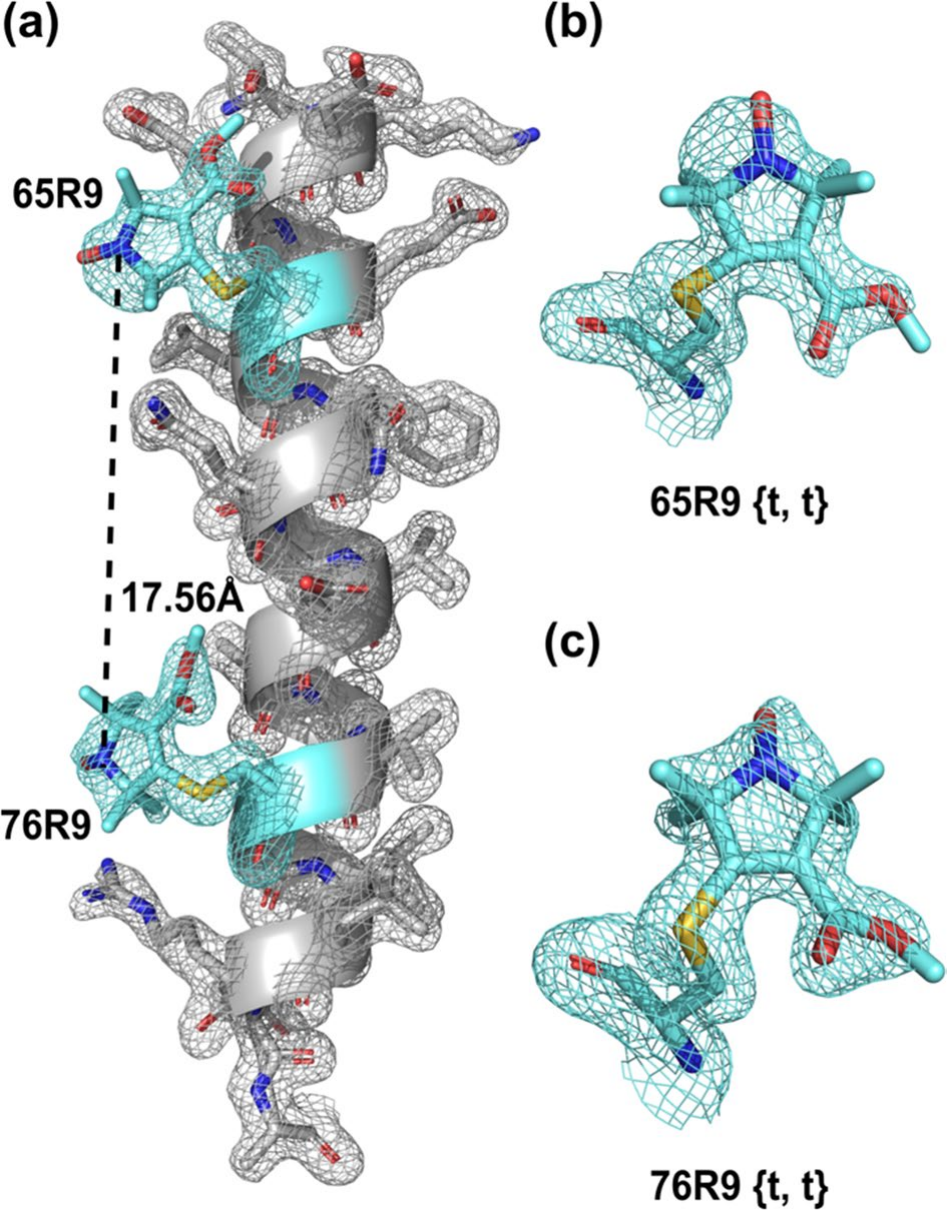
Crystal structure of T4L 65R9/76R9 at 100 K. **a** Electron density map for R9 and neighboring side chains are shown for the C helix. The electron density (cyan mesh) was calculated as an unweighted 2F_O_–F_C_ map contoured at 1.0 *σ*. The experimental interspin distance between 65R9 {*t*, *t*} and 76R9 {*t*, *t*} is indicated. **b** and **c** Electron density maps of 65R9 and 76R9, respectively. The structure is deposited to the RCSB Protein Data Bank (PDB code: 8TAT)

Figure 5 shows the electron density map for 65R9 and 76R9; electron densities of native side chains in Helix C are also shown to indicate the quality of the structure. Remarkably, the 2F_O_–F_C_ electron density map reveals a single conformation of the side chain at both sites 65 and 76, with nearly all non-hydrogen atoms resolved. This is consistent with the high order of R9 reflected in CW-EPR spectra. In contrast, electron density maps of the more mobile R1 at solvent-exposed helical sites are not resolved for the nitroxide ring due to disorder about the final two single bonds (*χ*_4_ and *χ*_5_) [5, 8].

Rotamers of a side chain with respect to dihedral angles {*χ*_1_, *χ*_2_} (Fig. 1) are designated *t* (trans), *p* (plus), or *m* (minus). For native amino acid side chains, nominal values are ± 180°, + 60° and − 60°, respectively, but variations of up to ± 30° are commonly observed [12, 43, 44]. For R1, V1, and RX, {*m*, *m*}, {*t*, *p*}, and {*t*, *m*}, are the predominant rotamers observed on *α*-helices in crystal structures [5, 8, 22, 25, 28, 45]. These rotamers are apparently stabilized by intra-residue interactions between the disulfide moiety and the peptide backbone, especially a C_α_-H···S_δ_ interaction [8]. Instead, the R9 side chain adopts a {*t*, *t*} conformation at both sites 65 and 76 (Table 1) that has not so far been observed in any structures of disulfide-linked spin labels at α-helical sites.

**Table 1:** Conformation of the T4L 65R9 and 76R9 side chains

Dihedral angle (*χ*) (°) or bond angle (∠)(°) or distance (*d*) (Å)	T4L 65R9 {*t*, *t*}	T4L 76R9 {*t*, *t*}
*χ*_1_	− 173.73	− 177.01
*χ*_2_	− 178.50	− 153.24
*χ*_3_	+ 136.46	+ 121.38
∠C_β_–S_γ_–C_3_	+ 94.25	+ 96.84
*d* (C_β_–S_γ_)	1.751	1.770

**Fig. 6:**
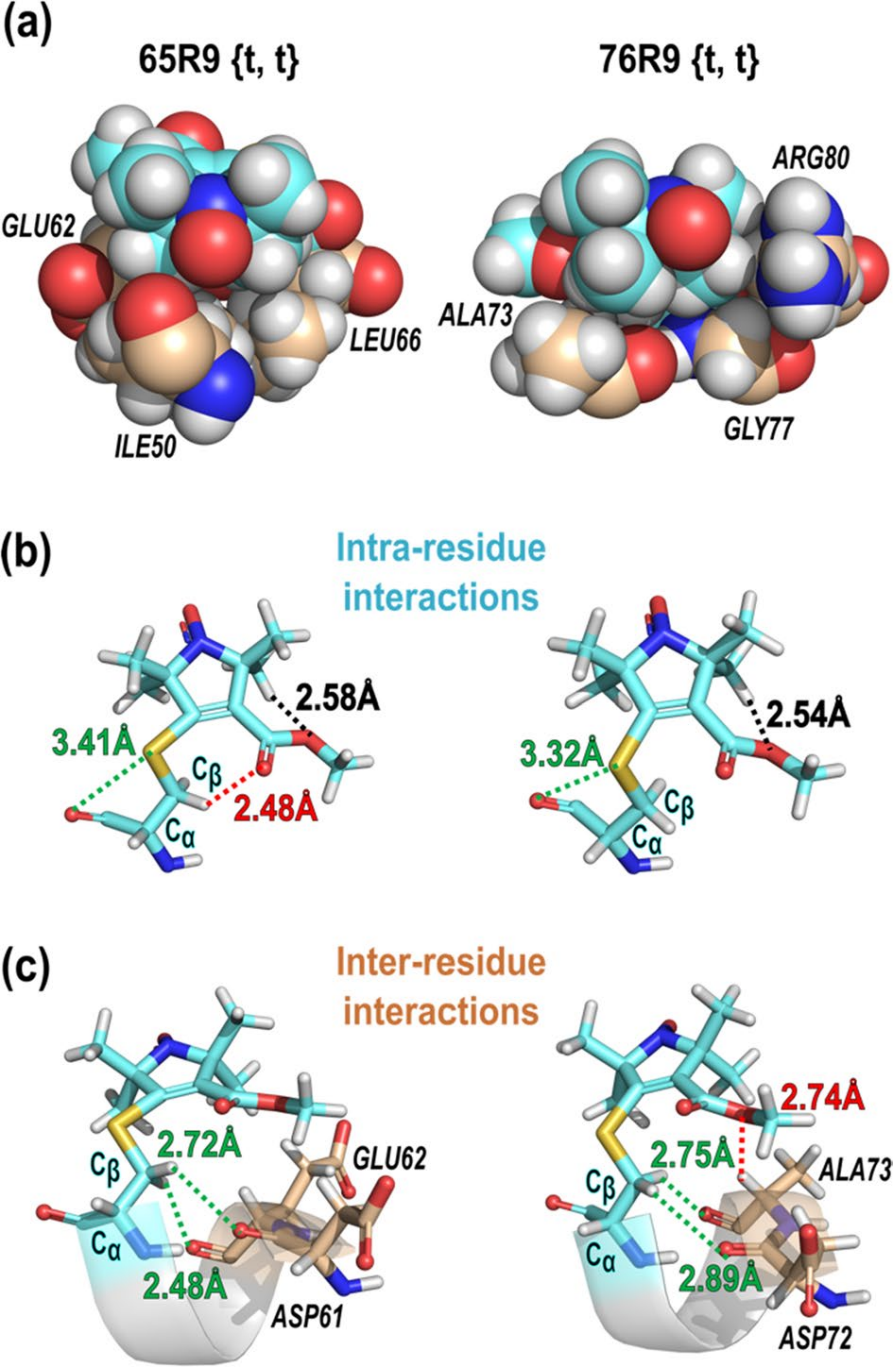
Interactions that stabilize the {*t*, *t*} rotamers and immobilize the nitroxides of R9. **a** CPK models of 65R9 {*t*, *t*} and 76R9 {*t*, *t*}. **b** Specific intra-residue, and **c** inter-residue interactions that stabilize the structure of 65R9 {*t*, *t*} and 76R9 {*t*, *t*} on Helix C determined from the crystal structure. In both panels, green dashes: interactions stabilizing the *χ*_1_ configuration as {*t*}; red dashes: interactions expected to hinder the motion of the nitroxide ring; black dashes: interactions restraining internally the configuration of the nitroxide ring

Due to the short linkage of the R9 side chain, the nitroxide ring resides close to the backbone in the {*t*, *t*} rotamer and is sufficiently closely packed to exclude solvent on one side of the ring. This suggests that non-specific hydrophobic/van der Waals interactions [46] may play a role in determining the rotamer and the nitroxide immobilization (Fig. 6a). In addition are more specific interactions that may be grouped as intra-residue interactions within the R9, or inter-residue interactions with the i-3 and i-4 residues.

Specific interactions that stabilize the *χ*_1_ configuration as {*t*} in both 65R9 and 76R9 are shown as green dashed traces in Fig. 6b and c, including intra-residue C=O···S_γ_ interactions that meet the distance and geometry criteria for *σ*-hole interactions [47–49] and inter-residue C_β_-H···O=C hydrogen-bonding interactions [50, 51] with backbone residues i-3 and i-4. These interactions stabilize the {*t*, *t*} rotamer, but do not constrain the motion of the nitroxide ring.

Interactions expected to hinder the motion of the nitroxide ring involve both intra- and inter-residue interactions of the 4-ester substituent and are shown as red dashed traces in Fig. 6. The plane of the ester function is tilted approximately 30°–35° relative to the plane of the nitroxide ring, a configuration that optimizes intra-residue hydrogen-bonding between the ester oxygen and the C_7_–H hydrogen (black traces, Fig. 6b). In addition, the inter-residue interaction of the ester oxygen with C_α_-H hydrogen of the i-3 residue is identified as potential C_α_-H···O H-bonds [50–55] (Fig. 6c). Collectively, these attractive interactions stabilize the position of the nitroxide ring and are apparently involved in the relative immobilization of R9 compared to R1.

The interactions identified above for the {*t*, *t*} rotamer do not depend on the identity of the nearest neighbor side chains beyond the C_β_ carbon. Thus, it is reasonable to assume that the above interactions, along with non-specific packing interactions, would be retained for R9 at helix surface sites in general. Under such conditions, the motion of the nitroxide would be modulated by collective motions of the helix, opening the possibility to utilize R9 as a sensor for slow protein dynamics, as discussed further below.

Although only the {*t*, *t*} rotamer is observed, other rotamers can be modeled with similar attractive interactions and without steric clashes (see below). Indeed, DEER distance measurements of the protein in frozen solution suggest the existence of at least one other preferred rotamer of R9. The results are presented in the following section.

### R9 Interspin Distance Measurements by Pulsed Dipolar EPR Spectroscopy

Double electron–electron resonance (DEER) spectroscopy under cryogenic temperatures is well established for measuring long-range distance (20–80 Å) between pairs of spin labels selectively introduced in proteins [21, 56–58]. An important feature of DEER is that it provides analytical probability distributions, instead of average distances, making this technique a powerful tool for revealing structural heterogeneity.

While the position of a particular peak in a distance distribution contains structural information, the width reflects the spatial disorder in both the protein and nitroxide side chains. Disorder in the protein at cryogenic temperatures may reflect structural fluctuations at physiological temperature and is thus of significant interest. For a distribution width to reflect protein disorder, the disorder in the side chain must be minimized. As shown above, the motion of the R9 side chain exhibits the most ordered motion observed for singly attached spin labels at the T4L 72 reference site, making it a candidate for investigating protein structural heterogeneity.

**Fig. 7:**
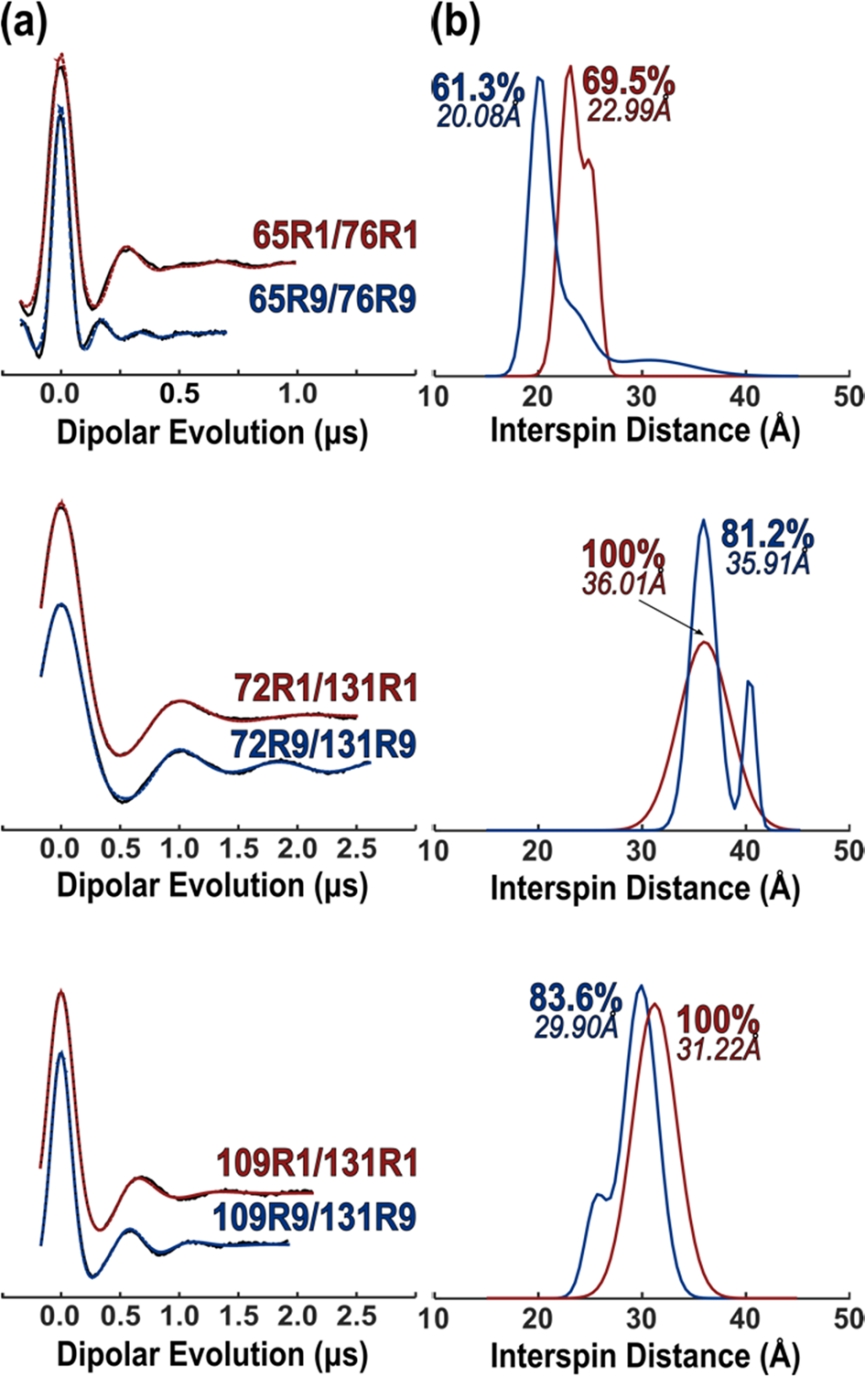
Interspin distances measured by DEER. **a** Background-subtracted dipolar evolutions of the indicated T4L mutants bearing either two R9 side chains (blue) or two R1 side chains (red). **b** Corresponding probability distance distributions obtained from fits using a Gaussian model (see SI). Similar distance distributions were obtained with model-free analysis using optimized Tikhonov regularization. Validations of the distance distributions are shown in Fig. S4

To evaluate this point, and discover other possible rotamers of R9, DEER measurements were made on three pairs of R9 labels introduced at T4L sites in regular helices. Analogous R1 spin pairs were prepared for comparison. The Dipolar evolution functions and the corresponding fits (Fig. 7a), and derived distance distributions (Fig. 7b) are shown in Fig. 7. For each R9 pair, the distance distributions (blue traces) are dominated by a narrow peak with full width at half maximum (FWHM) of 3–3.5 Å contributing 60–80% of the total probability, while the FWHM of the corresponding R1 pairs is 5–6 Å. Although the distance distributions of R9 contain satellite peaks, the narrow width of the main peak is consistent with the high order observed for the R9 side chain.

Interestingly, the most probable distance for the 65R9/76R9 (20.08 Å) is significantly longer than the interspin distance determined from the crystal structure (Fig. 5a, 17.56 Å). Assuming that the protein structure in the crystal and solution are the same, a rotamer of R9 other than {t, t} must dominate at one or both sites in the solution. The rotamer at 65R9 is the more likely candidate since R9 makes direct contact with a symmetry-related molecule in the crystal lattice that may stabilize {*t*, *t*} (Fig. S5), and such interactions would be absent in the solution.

**Fig. 8:**
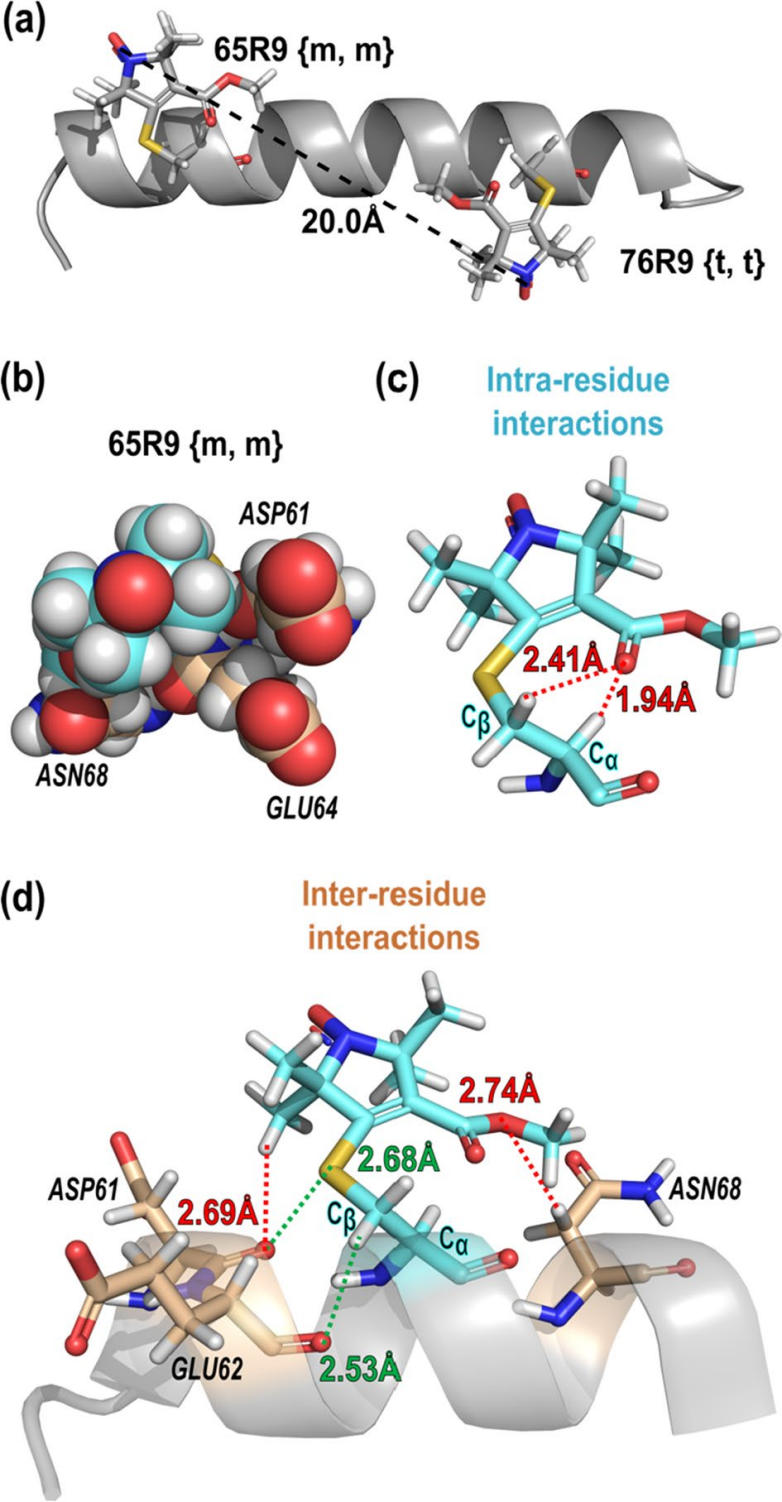
Models of the {*m*, *m*} rotamer at site 65 that account for experimental DEER interspin distance measurements. **a** Model of the Helix C with 65R9 {*m*, *m*} and 76R9 {*t*, *t*} showing the interspin distance; **b** CPK model of 65R9 {*m*, *m*}; **c** intra-residue and **d** inter-residue interactions that stabilize 65R9 {*m*, *m*}, respectively. In both panels, green dashes: interactions stabilizing the *χ*_1_ configuration as {*m*}; red dashes: interactions expected to hinder the motion of the nitroxide ring

Modeling indicates that the {m, m} rotamer, which can account for the observed 20.0 Å DEER distance between 65R9/76R9 (Fig. 8a), can be accommodated at site 65 without steric clashes (Fig. 8b). In modeling, the {m, m} rotamer would be stabilized by favorable attractive interactions, both intra- and inter-residue (Fig. 8c, d), including a unique H-bond interaction between the ester carbonyl oxygen and the acidic C_α_–H hydrogen [50–54]. For 72R9/131R9 and 109R9/131R9, the main distance peaks can be accounted for by sets of {*m*, *m*} and {*t*, *t*} rotamers (Fig. S6), but not with pairs of {*t*, *t*} rotamer. The minor distances observed in their distributions presumably arise from yet other rotamers.

### Spin–Lattice Relaxation Rate of R9

Nitroxide spin labels with highly ordered motions, such as R9, are expected to have long spin–lattice relaxation times (*T*_1_) [59–61], which are important for determining long-range distances at physiological temperatures using relaxation enhancement [62–64] or for measuring slow motion using ST-EPR [17, 65–67]. Based on CW-EPR lineshapes, it is anticipated that R9 should have a long *T*_1_ and therefore serve as a new probe for slow protein dynamics. To explore the suitability of R9 for those potential applications, *T*_1_ was measured using pulsed SR-EPR [29, 35, 36].

**Fig. 9:**
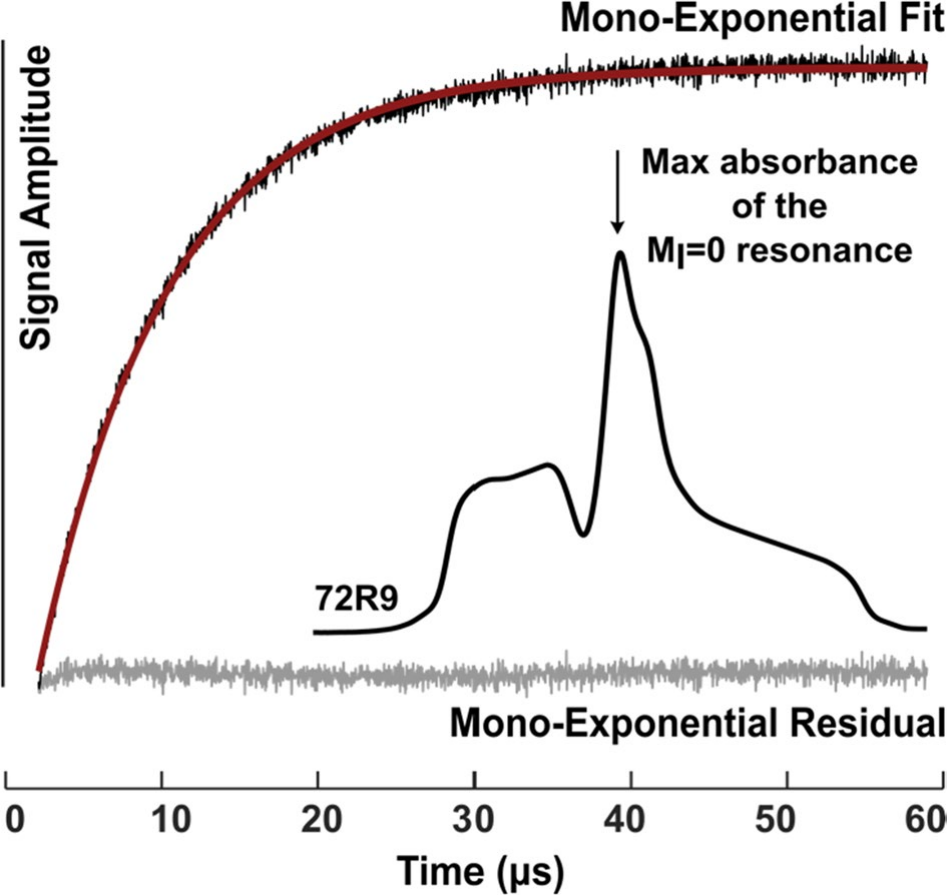
SR-EPR of T4L 72R9 attached to CNBr-Sepharose. The plot shows the signal intensity recorded at the nitroxide *m*_I_ = 0 resonance maximum in the absorption spectrum as a function of time, following a saturating microwave pulse delivered at the same position. The Saturation Recovery signal (black), monoexponential fit (red), and residuals (gray) are indicated

Figure 9 shows the recovery of the EPR signal from saturation for T4L 72R9 immobilized on CNBr-Sepharose. The recovery curve is reasonably well fit by a monoexponential function with a characteristic *T*_1_ = 8.3 μs, among the longest measured for a nitroxide spin-labeled protein at ambient temperatures [22]. With a *T*_1_ this long, it is predictable that R9 will be useful for relaxation enhancement experiments, extending the practical distance range up to ~ 45 Å [63, 64].

### Extending the Detection Range of Dynamics to the Microsecond Time Scale with R9

Although many functionally important processes of biological systems occur on the μs–ms time scale [68–73], a few spectroscopic techniques can offer direct dynamic information in this time domain. Conventional CW-EPR spectral lineshapes of nitroxides are insensitive to motions with correlation times longer than about 10^–7^ s. However, ST-EPR, a nonlinear continuous-wave EPR method introduced by Hyde and co-workers [16], extends the detection limit to motions with correlation times as long as milliseconds [17, 67, 74]. The technology has been used extensively to monitor the rotational diffusion of both soluble and membrane proteins [75–79].

To employ ST-EPR to observe slow internal modes of proteins, it is necessary to remove or minimize contributions from the overall Brownian protein diffusion and fast internal motions of the spin label. For this purpose, the protein is immobilized on a solid support [80, 81] and a spin label with restricted internal motion [81, 82] is needed. Here, we demonstrate that R9 is sufficiently immobilized on regular helix surface sites to detect microsecond dynamics in surface-attached proteins using ST-EPR.

**Fig. 10:**
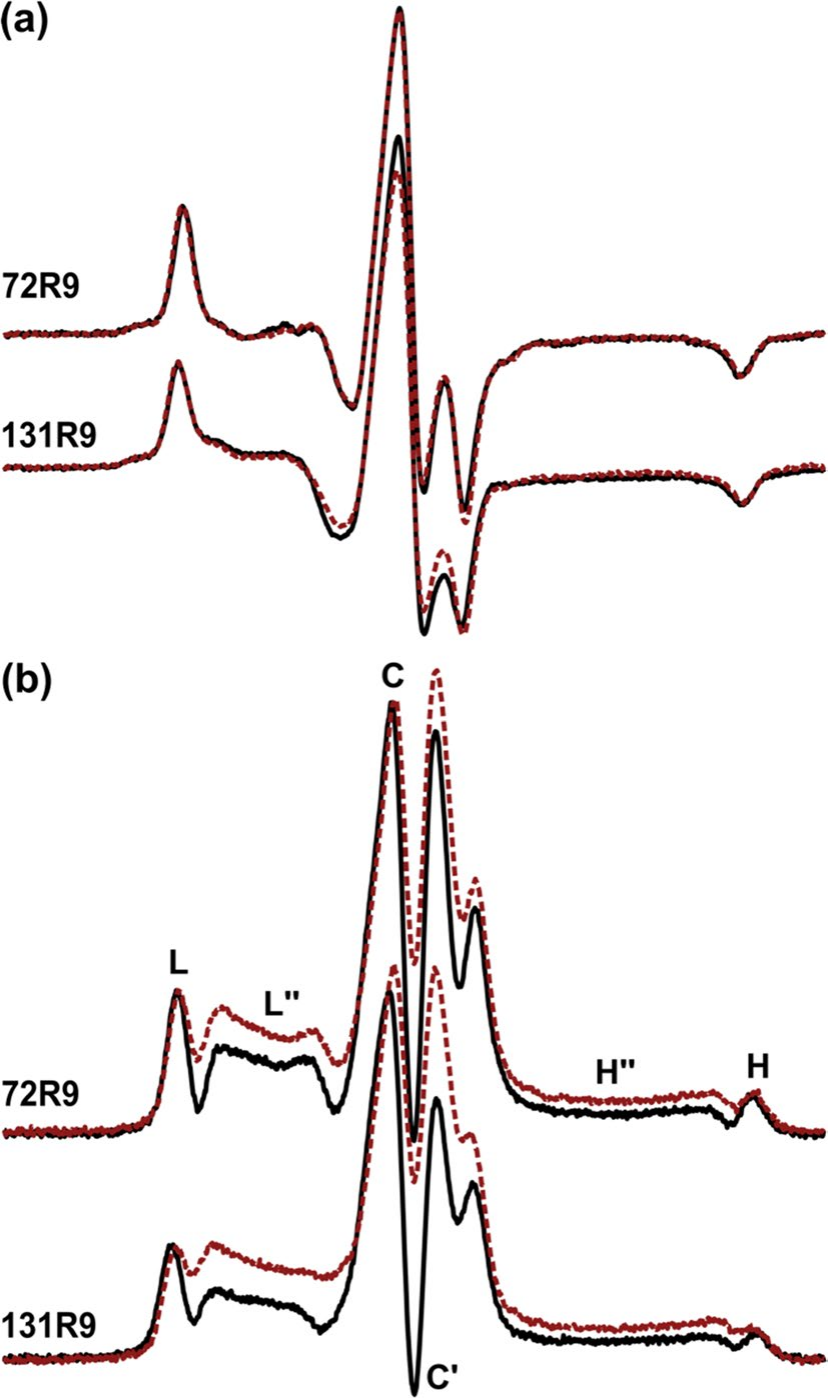
ST-EPR of T4L 72R9 and 131R9. **a** CW-EPR and **b** ST-EPR spectra of T4L 72R9 and T4L 131R9 attached to CNBr-Sepharose at room temperature, before (black, solid), and after (red, dash) crosslinking using glutaraldehyde. The following field positions for ST-EPR analysis are indicated: L is the resolved peak in the low field. L″ is defined as the line height at the field position 10 Gauss upfield of L. H is the resolved peak in the high field, and H″ is 15 Gauss downfield of H. The C and C′ are the first peak and trough in the center. All line heights are measured with respect to the baseline

Figure 10a and b (black traces) shows the CW-EPR and ST-EPR spectra, respectively, measured for T4L72R9 and T4L131R9 immobilized on CNBr-Sepharose at room temperature. Both CW-EPR lineshapes are similar and correspond to motion approaching the “rigid limit” for CW-EPR (*τ*_R_ ~ 100 ns). Information on motions in the μs and longer time domain is encoded in the line-height ratios [17, 74, 83, 84] H″’/H, C′/C, and L″’/L of ST-EPR spectra (Fig. 10b), using calibration plots [74] that relate the ratios to effective correlation times. The values of H″/H and L″’/L showing maximal sensitivity [17, 74] for motions in the time range of 10^–5^ ~ 10^–3^ s are used here (C′/C is sensitive when *τ*_R_ < 10^–5^ s).

The ST-EPR of 72R9 and 131R9 are also qualitatively similar, and values of L″’/L indicate motions with correlation times around 23 μs for each. On the other hand, the H″’/H ratio, which has higher sensitivity to slower motions, corresponds to ~ 50 μs and ~ 100 μs for 72R9 and 131R9, respectively. The data show the utility of R9 in ST-EPR to reveal slow dynamics well beyond the detection limit of conventional CW-EPR. The different values of *τ*_R_ derived from different line-height ratios indicate the anisotropic nature of the motion [83–87].

To reveal the origin of microsecond dynamics, labeled proteins on CNBr-Sepharose were treated with glutaraldehyde under conditions that create crosslinks between lysine residues within a protein [88]. This strategy has been widely used in both solid-state NMR [89–91] and EPR [76, 92] studies to immobilize proteins via inter-molecular crosslinks, although intramolecular links are simultaneously formed. In the present case where each protein molecule is individually tethered to the Sepharose and widely separated, intramolecular crosslinks dominate and are expected to modulate internal fluctuations of the protein, while the internal motion of the R9 side chain is unlikely affected.

Crosslinking with glutaraldehyde has little effect on the CW-EPR spectra of both sites (red dashed traces, Fig. 10a), but pronounced changes corresponding to more than the doubling of correlation times are evident in the ST-EPR spectra (red dashed traces, Fig. 10b) of both 72R9 and 131R9 (> 100 μs, > 200 μs, respectively, using the H″/H ratio), suggesting that internal protein dynamics are the origin of the slow motions detected by R9.

## Discussion

Elucidation of the sequence-specific nature of protein structure and dynamics is a primary goal of SDSL-EPR. Extracting such information from the EPR spectra of spin-labeled proteins requires knowledge of the configuration and motion of spin label side chain itself. Prior studies have thoroughly characterized the widely used nitroxide side chains R1, V1, and RX [3, 4, 8, 13, 15, 22, 25, 45, 93–95]. In this report, we provide a similar characterization of the new spin label side chain R9.

One important advantage of R9 is that it can be introduced with high selectivity to solvent-exposed cysteines. Presumably, this selectivity is due to the relatively slow reaction kinetics of HO-4072 with cysteine (Fig. 2b). Reaction with a buried cysteine can only occur during the lifetime of a protein fluctuation that exposes the residue to solvent, and the reaction rate of the Michael addition is too slow for a measurable product to form during the time required for reaction with a surface cysteine. On the other hand, the reactions of MTSSL and IDSL are extremely rapid, where quantitative modification with buried cysteines can occur [3].

An additional advantage of R9 is the thioether linkage that is not cleaved by the commonly employed reducing agents. Other spin label side chains linked to the protein via a thioether have been employed [81, 82, 96–101], but have not yet been characterized at the level required for interpreting EPR spectra in terms of protein structure and dynamics.

The CW-EPR lineshape of R9 at the T4L 72 reference site reflects a highly ordered anisotropic motion of the nitroxide (Fig. 3a), consistent with the very long *T*_1_ (Fig. 9) [61]. The high ordering of R9 is not unique to site 72 but is also observed at other sites in the interior of relatively rigid helical segments, including site 131, and sites 65 and 76 for which crystal structures were obtained (Fig. 5). The crystal structures suggest that the ordering arises from site-independent non-covalent bonding interactions with apparent contributions from non-specific packing interactions (Figs. 6, 8). On the other hand, the spectral lineshapes at most sites near helix termini and in connecting loops have increased mobility, suggesting that R9 may serve as a sensitive indicator of nanosecond motions in flexible regions of a protein (see below).

Highly constrained nitroxide side chains, such as RX and R9 with long T_1_, are of value in SDSL in part because they offer the potential for monitoring motions on the important μs–ms time scale, thus extending the detection range of conventional X-band CW-EPR lineshape analysis. To date, only methods relying on the saturation behavior of the nitroxide, determined by T_1_, have reported sensitivity to motions beyond the 100 ns time scale. Pulsed SR-EPR and ELDOR have been used to monitor exchange events in the range of 1 μs ~ 70 μs [29], while longer times are inaccessible. In principle, ST-EPR methods can extend the range farther into the ms domain [66, 67] provided that a highly constrained side chain is used and rotational diffusion of the whole protein can be minimized. Elegant studies by Thomas et al. have employed ST-EPR to monitor slow protein movements in muscle fibers as well as in lipid membranes, both of which provide a natural scaffolding on which labeled proteins are immobilized [18, 75, 76, 80, 81, 102–104]. Here, we extend this method to small (< 60 kDa), soluble proteins by immobilizing them on the Sepharose surface and employing R9 as the rigid probe.

The data in Fig. 10 demonstrate that ST-EPR using R9 can detect microsecond internal protein dynamics. The 20–100 μs motions detected for T4L 72R9 and T4L 131R9 attached on CNBr-Sepharose could in principle arise from motions of the protein relative to the solid support, R9 relative to the protein, and/or internal protein motions, i.e., structural fluctuations. The latter is clearly indicated by the strong effect of intramolecular crosslinking via glutaraldehyde. The ability to monitor site-specific internal motions might open the door to exploring the role of protein dynamics in ligand binding and protein–protein recognition, and applications will be presented elsewhere.

In addition to monitoring slow dynamics, R9 at highly ordered sites shows promise for distance measurement in proteins using DEER or T_1_-based relaxation enhancement methods (Fig. 7). For either method, an ideal label would exist in a single rotameric state to provide the highest distance resolution. It is unfortunate that multiple rotamers exist for any singly attached spin label published as yet, but due to the fewer number of rotatable bonds between the backbone and the nitroxide in R9 (3 bonds), as compared to R1 (5 bonds) and V1 (4 bonds) (Fig. 1), the rotameric space of R9 is more limited and the position of the EPR-active nitroxide is relatively localized.

For high-resolution structure mapping by DEER, the existence of multiple rotamers is a limitation, but the very narrow dominant population of interspin distances for R9 pairs suggests that R9 will be useful for assessing structural heterogeneity, particularly when monitoring backbone flexibility or small changes in the global structure. In general, the elucidation of equilibrium structure and dynamics can be improved by comparing distance distributions obtained with R1, V1, and R9 using DEER.

## Conclusions

Compared to the commonly used R1 side chain, the highly restricted internal motion of R9 makes measurement of slow motions within proteins possible, and allows for measurements of longer distances via relaxation enhancement at ambient temperatures and identification of structural heterogeneity via DEER. While the side chains V1 and RX have an ordering similar to R9, R9 has the advantages of labeling-selectivity at solvent-exposed surface sites, and singly attachment via a non-reducible thioether linker. Each of these side chains has advantages and disadvantages, but the unique properties of R9 make it a useful addition to the SDSL toolkit.

## Materials and Methods

### Construction, Purification, and Spin Labeling of Cysteine Mutants

T4L constructs were expressed, purified, and spin-labeled according to the general procedures previously described [3, 22, 25]. Reagents MTSSL and HO-1944 were generous gifts of Prof. Tamás Kálai (University of Pécs, Hungary). IDSL was purchased from Toronto Research Chemicals. Additional information is in SI Materials and Methods.

### Synthesis, Characterization, and Reaction Kinetics of Reagent HO-4072


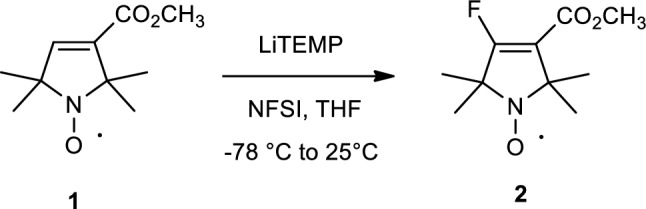


The synthesis of Methyl 4-fluoro-2,2,5,5-tetramethyl-2,5-dihydro-1H-pyrrole-3-carboxylate-1-yloxyl Radical (HO-4072): To a stirred solution of 2,2,6,6-Tetramethylpiperidine (TEMP) (1.51 g, 10.0 mmol) in anhydrous THF (10 mL), BuLi 4.0 mL (10.0 mmol, 2.5 M in hexanes) was added at 0 °C to generate the Li salt of TEMP (LiTEMP). After stirring at this temperature for 20 min. the solution was chilled to − 78 °C with dry ice/acetone and the solution of compound **1** [105] (1.98 g, 10.0 mmol) in anhydr. THF (10 mL) was added dropwise during 30 min. After stirring this solution at − 78 °C for 1 h, fluorobenzosulfonimide (NFSI) (3.15 g, 10.0 mmol) was added in anhydr. THF (20 mL) at − 78 °C during 30 min. Then, the mixture was allowed to warm to room temperature spontaneously (~ 1 h). The mixture was quenched with aq. sat. NH_4_Cl solution (20 mL) followed by adding EtOAc (20 mL). The organic phase was separated, then the aqueous phase was extracted with EtOAc (20 mL), and the combined organic phase was dried (MgSO_4_), filtered, and evaporated. The residue was purified with flash chromatography (hexane/ Et_2_O) to give compound **2** 583 mg (27%) as orange crystals.

Compound **2** (reagent HO-4072) was characterized by chromatography and multiple spectroscopies. The kinetics of the reaction were determined using EPR spectroscopy, and the reaction products were characterized by Electrospray Ionization Mass Spectrometry (ESI–MS). Details are described in SI Materials and Methods.

### EPR Spectroscopy

CW-EPR and ST-EPR experiments were performed on a Varian E-109 spectrometer fitted with a two-loop one-gap resonator [106, 107]. SR-EPR and Four-pulse DEER experiments were conducted on the Bruker ELEXSYS 580 spectrometer. Spectral simulations and DEER data analyses were carried out using the programs “MultiComponent” and “LongDistances”, respectively. The programs are available at https://www.biochemistry.ucla.edu/Faculty/Hubbell/software.html. Additional details in *SI Materials and Methods*.

### X-Ray Crystallography

The diffraction quality crystal of T4L 65R9/76R9 was grown using the hanging drop vapor diffusion method. Crystals appeared in 1 week under 4℃. For the cryogenic diffraction study, a single crystal cryo-protected in mineral oil was flash-frozen, and data were collected at 100 K using an FR-E + SUPERBRIGHT X-ray Generator on a RIGAKU RAXIS HTC image plate detector. Data processing was performed as previously described [8] (SI Materials and Methods).

### Supplementary Information

Below is the link to the electronic supplementary material.

Supplementary file1 (DOCX 1673 kb)

## Data Availability

The data that support the findings of this study are available from the corresponding author upon reasonable request.

## Declarations

### Data Deposition

The atomic coordinates and structure factors for T4L 65R9/76R9 have been deposited in the Protein Data Bank, www.pdb.org (PDB ID code 8TAT).
