# Supplementary material for: A Highly Ordered Nitroxide Side Chain for Distance Mapping and Monitoring Slow Structural Fluctuations in Proteins
Source: Appl Magn Reson. 2023 Oct 14;55(1-3):251–77. doi: 10.1007/s00723-023-01618-8 (PMC10861403; doi:10.1007/s00723-023-01618-8)
Supplement: Supplementary file 1 — Supplementary file1 (DOCX 1673 kb) [file 723_2023_1618_MOESM1_ESM.docx]

**Supplementary Information**

**A Highly Ordered Nitroxide Side Chain for Distance Mapping and Monitoring Slow Structural Fluctuations in Proteins**

**Mengzhen Chen^1^, Tamás Kálai^2^, Duilio Cascio^3^, Michael D. Bridges^1^, Julian P. Whitelegge^4^, Matthias Elgeti^1,5^, Wayne L. Hubbell^1^**

^1^Jules Stein Eye Institute and Department of Chemistry and Biochemistry, University of California, Los Angeles, CA 90095, USA

^2^Institute of Organic and Medicinal Chemistry, Faculty of Pharmacy, University of Pécs, Szigeti St. 12, H-7624 Pécs, Hungary

^3^Departments of Chemistry and Biochemistry, University of California, Los Angeles, UCLA- Department of Energy Institute for Genomics and Proteomics, Molecular Biology Institute, Howard Hughes Medical Institute, CA, 90095, USA

^4^The Pasarow Mass Spectrometry Laboratory, David Geffen School of Medicine, The Jane and Terry Semel Institute for Neuroscience and Human Behavior, University of California, Los Angeles, CA, 90095, USA

^5^Present Address: Institute for drug discovery, Leipzig University Medical Center, Härtelstr. 16-18, 04107 Leipzig, Germany

Corresponding author: Wayne L. Hubbell, [hubbellw@jsei.ucla.edu](mailto:hubbellw@jsei.ucla.edu)

**Materials and methods**

**Construction, Purification, and Spin Labeling of Cysteine Mutants**

T4L constructs were expressed, purified, and spin-labeled with MTSSL, IDSL, or HO-1944 as previously described [1–3]. Briefly, all mutants were generated by QuickChange site-directed mutagenesis of the pET11a-T4L; mutations were verified by DNA sequencing. All T4L cysteine mutants contain the pseudo-wild-type mutations C54T and C97A [4–6]. The purified and desalted cysteine mutants (in 50 mM MOPS, 25 mM NaCl, pH = 6.80) were subjected for 1h at room temperature to a 10-fold molar excess of MTSSL or IDSL; for HO-1944 a one- to three-fold molar excess was used instead to avoid double-labeling. The reactions were allowed to proceed overnight at room temperature.

For generating the thioether-linked side chain R9 with HO-4072, the sample was desalted into a buffer consisting of 10 mM MES, 100 mM NaCl, pH 6.0. To the desalted protein was added a 10-fold molar excess of HO-4072 (as 100mM in acetonitrile) and the reaction was initiated by adjusting the pH to 8~9 with appropriate volumes of high pH buffers (250 mM Bis-tris propane or 250 mM sodium borate, 100 mM NaCl, pH = 11).

The excess spin-labeling reagent was removed by desalting using a HiTrap (GE Healthcare) desalting column. The spin-labeled protein was then concentrated using an Amicon Ultra centrifugal filter Device (10 kDa cutoff) (Millipore, Bedford, MA) and stored until use. Corresponding mutants were covalently attached to CNBr-activated Sepharose 4B (GE Healthcare) as previously described for R1 mutants [7, 8].

**Characterization and reaction kinetics of spin labeling reagent HO-4072**

*Characterization of spin labeling reagent HO-4072*

The compound HO-4072 was characterized by Mass spectrometry on an Automass multi-mass spectrometer operated in EI mode (70 eV). Elemental analyses were measured on a Fisons EA 1110 CHNS elemental analyzer and melting points were determined on a Boetius micro-melting point apparatus. IR spectra were obtained using a Bruker Alpha FT-IR instrument with an ATR accessory on a diamond plate. Flash column chromatography was performed on Merck Kieselgel 60 (0.040-0.063 mm). Thin-layer chromatography was run on Merck Kieselgel 60 F254. Compound **1** (Figure in the main text, Material and methods) was prepared as reported previously [9], all other reagents and solvents were purchased from Merck.

Results: Melting point (mp): 45-47 °C, R_f_: 0.74 (hexane/Et_2_O, 2:1). IR: 𝜈_max_ 2988 cm^-1^, 1715 cm^-1^, 1676 cm^-1^. Anal: calcd. For C_10_H_15_FNO_3_, C: 55.55; H: 6.99; N: 6.48; found: C: 55.51, H: 7.06, N: 6.31. MS (EI) m/z, (%): 216 ([M]^+^, 5), 186 (44), 154 (23), 123 (50), 73 (100).

*Monitoring potential ester hydrolysis of a thioether analogue of the R9 side chain, HO-5135 (Fig. S2)*

To test for ester hydrolysis, the thioether HO-5135 was solubilized in a mixture of acetonitrile (17 mL) and an aqueous buffer (83 mL). The buffer (2.4 cm^3^, 96% acetic acid, 2.7 cm^3^, 85% H_3_PO_4_, and 2.47 g boric acid in 1 L water) was adjusted to pH 8.7 with 0.2 M NaOH. The mixture was monitored by HPLC on a Hypersil BDS-C18 reversed-phase column (5 µm particle size, 4.6 mm inner diameter, 150 mm length) at a flow rate of 1 ml/min; a variable wavelength detector at 270 nm was used to detect HO-5135 and HO-5136 in acetonitrile. The mobile phase was a mixture of acetonitrile and deionized water at a volume ratio of 50:50 in an isocratic elution mode. Retention times (Fig. S2): 1.6 min HO-5136 and 6.5 min HO-5135.

*ESI Mass Spectroscopy for R9-labeled T4L*

In general, the purity of R9-labeled T4L samples for MS measurements was greater than 95 % as judged by SDS-PAGE electrophoresis after purification over the ion-exchange column. To ensure that there were no misfolded soluble-oligomeric species in the sample, the labeled-protein fraction was injected into a Superdex 75 (GE Healthcare) gel filtration column pre-equilibrated with 50 mM MOPS and 25 mM NaCl at pH 6.8. In all cases, only the peak with a retention volume of ∼15.1 mL corresponding to monomeric T4L was collected and measured.

Mass spectra were recorded using an electrospray ion-trap mass spectrometer (LTQ; Thermo Fisher) operated in positive ion mode. Protein samples (~3 μg protein in 10 μL buffer) were diluted in 40 μL formic acid (90%; ACS grade) with 30 sec vortex mixing prior to immediate loading to HPLC. Size-exclusion HPLC was performed at 40 °C using a silica column matrix (SW2000, 4 mm x 30 cm; Tosoh Biosciences) equilibrated in chloroform/methanol/1% formic acid in water (4/4/1; v/v) at 250 μL/min [10]. Protein eluted at around 7 minutes and the ESI mass spectrum was deconvoluted with MagTran software.

*Reaction Kinetics*

To determine the pseudo first-order kinetics of the reaction of HO-4072 with T4L 72C, the HO-4072 reagent (100 mM stock solution in acetonitrile) was added to a large molar excess of protein (15-fold or more) which was initially in a buffer at pH 6 (10 mM MES, 100 mM NaCl), and the pH adjusted to the desired final value (7 to 9) with appropriate volumes of high pH buffer (pH 11, 250 mM sodium borate, 100 mM NaCl). The rate of reaction was continuously monitored by the decrease in intensity of the narrow high field resonance line of the spectrum (inset, Fig. 2b), I_(t)_, which is directly proportional to the concentration of unreacted HO-4072. The high field resonance was used because the spectral overlap of the narrow component corresponding to the unreacted reagent, and the broad component corresponding to the protein-bound side chain is minimal [11, 12].

The rate of the reaction of the reagent HO-4072 (SL) with a cysteine anion on the protein (PS^-^) under pseudo first-order conditions, where the concentration of cysteine anion, [PS^-^] is approximately constant during the reaction, is given by:

$R= -\frac{d\left[ \mathrm{SL} \right]}{\mathrm{dt}}=k_{2}\cdot\left[ \mathrm{SL} \right]\cdot\left[ PS^{-} \right]\cong k_{1}^{'}\cdot[SL]$ (Eq. 1)

where R is the reaction rate, k_2_ is the true second-order rate constant, k_1_’ is the pseudo first-order rate constant, $k_{1}^{'}=k_{2}\cdot[\mathrm{PS}^{-}]$. Thus,

$\frac{R(t)}{R\left( 0 \right)}=\frac{I_{(t)}}{I_{(0)}}=e^{-k_{1}^{'}t}$ (Eq. 2)

where I_(t)_ is the EPR spectral intensity of unreacted HO-4072. The disappearance of the limiting HO-4072 is exponential in time and fits of the data directly provide the value of k_1_’ (Fig. 2b) at any constant pH. Since [PS^-^] depends on pH, so does k_1_':

$k_{1}^{'}=k_{2}\cdot\left[ \mathrm{PS}^{-} \right]=k_{2}\cdot\left[ P_{o} \right]\cdot({K_{a}}/{K_{a}+{10}^{-\mathrm{pH}}})$ (Eq. 3)

where [P_0_] is the total protein concentration [P_0_] = [PS^-^]+[SH], and K_a_ is the equilibrium dissociation constant of cysteine in the protein.

Experimental data for k_1_’ vs pH can be fit to Eq. 3 to yield the value of k_2_⋅[P_0_] (both pH independent) and the value of K_a_. With known concentrations of protein (determined by the UV_280_ absorbance; extinction coefficient 24750 M^-1^cm^-1^), the true second-order reaction rate constant, k_2_, can also be calculated. The pK_a_ of the single solvent-exposed cysteine can thus be estimated by fitting the pH dependence of rate constants to Eq.3.

**EPR Spectroscopy**

*CW spectroscopy*

EPR spectroscopy was performed on a Varian E-109 spectrometer fitted with a two-loop one-gap resonator [13, 14]. Samples of 5 μL (∼100-300 μM) were loaded in 0.6 mm I.D. x 0.84 mm O.D. glass capillary tube (VitroCom Inc, N.J.) sealed on one end. All CW-EPR spectra were acquired using a 2 mW incident microwave power and *ca.* 1 G field modulation amplitude at 100 kHz.

*Simulation of CW-EPR spectra*

EPR spectra were fit to the MOMD model of Freed and co-workers [15–17] using a modified Levenberg-Marquardt algorithm as implemented in a custom program, MuitiComponent, written by Christian Altenbach (Stein Eye Institute, UCLA) in LabVIEW (National Instruments, Austin, TX), software available online (<https://www.biochemistry.ucla.edu/Faculty/Hubbell/software.html>). A detailed description of the model and simulation parameters can be found in Budil et al [18]. A brief description specific to the implementation used in the present work is given below. For consistency, the notation used is that of Budil et al.

To fit the experimental spectra to the MOMD model, principal values of the nitroxide g- and A-tensors in the absence of motion (frozen solutions) in the appropriate solvent are required. For the g-tensor principal elements (g_xx_, g_yy_, g_zz_), published values for MTSSL in frozen aqueous solution were used [19]. For X-band EPR, small variations in g-tensor values for the different nitroxide derivatives do not significantly influence the fits and were fixed at the reported values, appropriate for a nitroxide surface residue in the aqueous solution.

For the hyperfine tensor, the important principal value A_zz_ was determined directly from the wide spectral splitting 2A_zz_’ in frozen solutions at liquid nitrogen temperature. The rather small and similar values for A_xx_ and A_yy_ were not well-determined from the broad frozen solution spectra at X-band. Instead, values of A_xx_ and A_yy_ were estimated from spectral simulations of the nitroxides undergoing anisotropic motion at room temperature, for which the spectral features are better resolved. Starting values for A_xx_ and A_yy_ were obtained from A_zz_ and the isotropic hyperfine coupling, A_iso_, where:

${A_{\mathrm{iso}}=1}/3(A_{\mathrm{xx}}+A_{\mathrm{yy}}+A_{\mathrm{zz}})$ (Eq. 4)

and

$3A_{\mathrm{iso}}-A_{\mathrm{zz}}=A_{\mathrm{xx}}+A_{\mathrm{yy}}$ (Eq. 5)

Values of A_iso_ are directly measured for spin labeling reagents in the aqueous solution (Table. S1). Based on the approximation of axial symmetry for the hyperfine tensor [20–22], starting values of A_xx_ ≈ A_yy_ were obtained from A_iso_ and Eq. 5.

The fit for the spectrum of each side chain in T4L attached to CNBr-Sepharose at room temperature (Fig. 3a) was first achieved by fixing the principal values of A and g tensors as determined above, and varying the rotational diffusion rates (R-tensors) and ordering potential coefficients (c_20,_ c_40_, c_42_, and c_44_), from which the correlation times (τ_R_) and the order parameters (S_20_) were derived. A_xx_ and A_yy_ were then allowed to vary to optimize the fit, if necessary, by small amounts from the starting values.

Finally, the optimized principal values of A, g, and R-tensors were fixed, only the ordering potential (c_20_), Gaussian inhomogeneous broadening (Δ^(0)^ and Δ^(2)^), and the Lorentzian linewidth tensor elements (W_xx_, W_yy_, W_zz_) were allowed to vary to improve the fits. Satisfactory fits could be obtained for RX, R9, V1, and R1 at the same effective correlation time τ_R_, with differences only in the order parameter S_20_, as shown in Fig. 3, where:

$\tau_{R}=\frac{1}{6<R>}$ (Eq. 6)

$<R>=\sqrt[3]{R_{\mathrm{xx}}R_{\mathrm{yy}}R_{\mathrm{zz}}}$ (Eq. 7)

The final values of the magnetic and other relevant parameters are given in Table. S1. In the case of V1, the ordering potential was more complex, and required coefficients c_40_, c_42_, and c_44_, in addition to c_20_.

*ST-EPR*

For ST-EPR, the spectra (V_2_’) were acquired using a 1.25 mW incident microwave power at which the strength of the magnetic field, H_1_, equals 0.25 G for this resonator (calibrated using the peroxylamine disulfonate in deoxygenated solution [23]). The field was modulated at 50 kHz with a modulation amplitude of 5 G. The reference phase on the phase-sensitive detector was carefully set to an approximate null under the unsaturated (H_1_ = 0.032 G), in-phase settings (V_1_) for each sample. Detailed calibration procedures, instrumental parameter settings, and reference curves for acquiring apparent correlation times can be found in previous publications [23–25].

*SR-EPR*

Saturation recovery EPR was performed on a Bruker E580 pulse bridge using a 2-loop-1-gap resonator, Stanford signal amplifiers, and the basic procedures as described elsewhere [26] with some minor modifications. In brief, the experimental temperature was controlled using a Bruker temperature controller and liquid nitrogen boiler. Approximately 5 μL of T4L 72R9 attached to CNBr-Sepharose was loaded into a TPX capillary with an I.D. of 0.6 mm (Molecular Specialties Inc., Milwaukee, WI), and before each measurement, the sample was equilibrated at 298 K under nitrogen flow to remove paramagnetic oxygen.

The saturating pulse length was 2 μs, with an incident power of 250 mW; the observing power was 100 μW. Both the saturating and observing pulses were set to the maximum absorbance of the nitroxide absorbance spectrum (i.e., the centerline absorption maximum). A shot repetition time (SRT) of 56 μs was used for each SR curve acquisition. Each SR curve was acquired with 2048 points at a 30 ns step size, and an analog bandwidth of 20 MHz. Typically, 1,048,576 accumulations were acquired per curve on- and off-resonance using a downfield step of 45 G at 0.5 Hz. 5 separate SR curves were acquired, phase corrected, and averaged as previously described [26], following simple off-resonance background subtraction of each. The total number of acquisitions for the SR curve reported herein was 5.3 million.

*DEER Spectroscopy*

Four-pulse DEER experiments for the double spin-labeled proteins were conducted at 80 K on the Bruker ELEXSYS 580 spectrometer equipped with a SuperQFT Q-band bridge and a 150-W TWT amplifier (Applied Engineering Systems, Fort Worth, TX), and fitted with an ER 5106QT-2 Q-band resonator (Bruker Siospin). Protein concentration for DEER samples was approximately 50-100 μM for all samples. 20 μL samples in D_2_O buffer containing 10-20% v/v deuterated-glycerol as cryoprotectant were loaded into quartz capillary tubes (2.0 mm I.D. x 2.4 mm O.D.; VitroCom Inc., NJ) and then flash-frozen in liquid nitrogen. Pulse lengths were optimized via nutation experiment as 16 ns (π/2) and 32 ns (π). Observer frequency was set to a spectral position 2 G downfield of the centerline resonance minimum turnover point, and the 100 ns π-pump envelope frequency was a 50 MHz-wide square-chirp pulse (generated by a Bruker arbitrary waveform generator) set 80 MHz downfield from the observer frequency. Additional parameters were set to the following values: d1 = 300 ns, d2 = variable (1500-3000 ns), d3 = 100 ns, d30 = 4 ns (only for 65R9/76R9) or 16 ns, d31 = 16 ns, phase cycling = 16-step.

Distance distributions were obtained by analyzing the raw dipolar evolution time raw data using LongDistances, a custom program written by Christian Altenbach (Stein Eye Institute, UCLA) in LabVIEW (National Instruments, Austin, TX), software available online (https://www.biochemistry.ucla.edu/Faculty/Hubbell/software.html). A dipolar evolution function was obtained after an exponentially decaying background function was applied to correct for random inter-molecular dipolar interactions. Processing of dipolar evolution data yields distance probability distributions that reflect all interacting spins within the 15-80 Å range [27]. DEER data were analyzed using a linear combination of model dipolar evolutions, with the linear coefficients constrained to a mixture of Gaussian distributions of variable mean, width, and amplitude. The most parsimonious model was determined by comparing the goodness of fit (chi-square) values for several fits using different numbers of Gaussians.

For the validation of distance distributions, the model-free distance distributions and determination of confidence intervals (as mean distance ±1 or 2 standard deviation σ, accumulation 10000) were also generated by the program LongDistances (details about calculating the mean distance and standard deviation can be found in: <https://sites.google.com/site/altenbach/labview-programs/epr-programs/long-distances/longdistances-online-help/ld-errors?authuser=0>). The algorithm in LongDistances is an optimized Tikhonov regularization that gives distributions equivalent to that used in other published analysis programs [28]. Smoothness values for each fit were chosen based on the calculated Tikhonov L curve.

**X-ray Crystallography**

T4L 65R9/76R9 was crystallized using the hanging drop vapor diffusion method. Reservoir buffers were prepared in twelve different concentrations of the precipitant (1.85-2.40 M Na/KPO_4_) all having 150 mM NaCl and 100 mM 1,6-hexanediol at eight different pHs (6.7-7.4) with or without the additive 3% 2-propanol. Crystallization trials were prepared by mixing purified labeled protein (~15 mg/mL in 50 mM MOPS, 25 mM NaCl, pH 6.8) with reservoir buffer by ratios of 1:2, 1:1, and 2:1, and by suspending the drops over 100μL of the same buffer. The final crystallization condition was 2.35 M Na/KPO_4_, pH 7.4 with 3% 2-propanol. Crystals appeared in 1 week under 4 ℃.

The crystals of T4L 65R9/76R9 were cryo-protected using mineral oil and were flash-frozen at 100 K with a nitrogen gas stream. Data were collected at 100 K using an FR-E+ SUPERBRIGHT X-ray Generator on a RIGAKU RAXIS HTC image plate detector, processed with XDS and XSCALE [29]. T4L 65R9/76R9 crystals grew in space group P3_2_21 and were isomorphous with the pseudo-wild type T4 Lysozyme crystals with similar cell dimensions [30]. The data sets showed only one molecule in the asymmetric unit. The structure was determined by molecular replacement method using PHASER [31]. PDB accession code 5JDT was used as a starting model. A randomly selected 5% of the data was set aside before the start of the refinement to calculate R_free_. Models were refined using PHENIX [32] and rebuilt using COOT [33]. The refinement quality was monitored using the R_free_. X-ray data collection and refinement statistics are reported in Table. S2, respectively.

**Protein immobilization using glutaraldehyde crosslinking**

5 μL of R9-labeled T4L attached to CNBr-Sepharose (protein concentration *ca.* 500 μM) was incubated with > 25-fold molar excess of ice-cold glutaraldehyde solution (Electron Microscopy Sciences, Hatfield, PA) at 4 ℃. The mixture was immediately loaded into a sealed 0.6 mm I.D. x 0.84 mm O.D. glass capillary tube (VitroCom Inc, N.J.), and subjected to quick centrifugation at 800 rpm for 1 minute to facilitate the settlement of beads at the bottom of the capillary tube. The crosslinking reaction was allowed to proceed at room temperature overnight. The crosslinked mixture formed a yellowish color over the beads after ~15 minutes of incubation time.

**Figures and Tables**

**Table. S1** Parameters for CW-EPR simulations^a,b^

|  | $\mathbf{A}_{\mathbf{xx}}$/G | $\mathbf{A}_{\mathbf{yy}}$/G | $\mathbf{A}_{\mathbf{zz}}$/G | $\mathbf{A}_{\mathbf{iso}}$/G | <R>/s^-1^ | τ_R_/ns | S | c_20_ |
| --- | --- | --- | --- | --- | --- | --- | --- | --- |
| 68RX72 | 6.31 | 5.40 | 36.68 | 16.12 | 7.85 | 2.35 | 0.926 | 13.69 |
| 72R9 | 5.73 | 5.30 | 36.29 | 15.77 | 7.85 | 2.35 | 0.859 | 7.51 |
| 72V1 | 6.23 | 5.51 | 34.97 | 15.57 | 7.85 | 2.35 | 0.669^c^ | 1.90 |
| 72R1 | 6.12 | 5.18 | 37.09 | 16.13 | 7.85 | 2.35 | 0.451 | 2.06 |

^a^The magnetic parameters used for simulations [19] are as follows:

$g_{xx}=2.00800, g_{yy}=2.00586, g_{zz}=2.00199$;

^b^$<R>=\sqrt[3]{R_{xx}R_{yy}R_{zz}}$ is log10 of the average rotational diffusion tensor [18];

^c^For the simulation of 72V1, c_40_ = 2.76, c_42_ = 2.23, c_44_ = 0.27 were required [3].

**Table. S2** X-ray data collection and refinement statistics for the T4L 65R9/76R9 crystal^a^

| **Data Collection** |  |
| --- | --- |
| PDB ID | **8TAT** |
| Temperature, K | 100 |
| Wavelength, Å | 1.5418 |
| Resolution, Å | 52.30-1.60 |
| Highest resolution shell, Å | 1.64-1.60 |
| Space group | P3_2_21 |
| Reflections |  |
| *Total* | 540000 (36935) |
| *Unique* | 50346 (3627) |
| *R_sym_, %* | 8.2 (96.2) |
| *Completeness, %* | 97.8 (95.5) |
| *Redundancy* | 10.7 (10.2) |
| *I/σ* | 16.04 (2.68) |
| Unit cell dimensions |  |
| *a, Å* | 60.39 |
| *b, Å* | 60.39 |
| *c, Å* | 95.43 |
| **Refinement** |  |
| Resolution, Å | 52.30-1.60 |
| Highest resolution shell, Å | 1.66-1.60 |
| Reflections |  |
| *Used* | 26611 (2434) |
| *Completeness, %* | 97.64 (96.00) |
| ^b^R_work_, % | 19.77 (21.91) |
| ^c^R_free_, % | 21.85 (23.09) |
| R. M. S. deviations |  |
| *Bond lengths, Å* | 0.006 |
| *Bond angles, °* | 0.874 |
| Mean B-factor, Å^2^ |  |
| *Protein atoms* | 23.0 |
| *Non-protein atoms* | 28.7 |
| Validation statistics |  |
| *Ramachandran analysis, Favored, %* | 98.70 |
| *Ramachandran analysis, Allowed, %* | 1.30 |
| *Ramachandran analysis, Outliners, %* | 0.00 |
| *MOLPROBITY Clashscore* | 2.31 |
| *Estimated coordinate error, Å* | 0.15 |

^a^ Statistics for the highest-resolution shell are shown in parentheses.

^b^ R_work_ = $(\sum\left| F_{O}-F_{C} \right|/\sum\left| F_{O} \right|)\times100$, where F_O_ is the observed structure factor amplitude and F_C_ is the calculated structure factor amplitude.

^c^ R_free_ is R_work_ calculated using 5% of the data, randomly chosen and omitted from refinement.


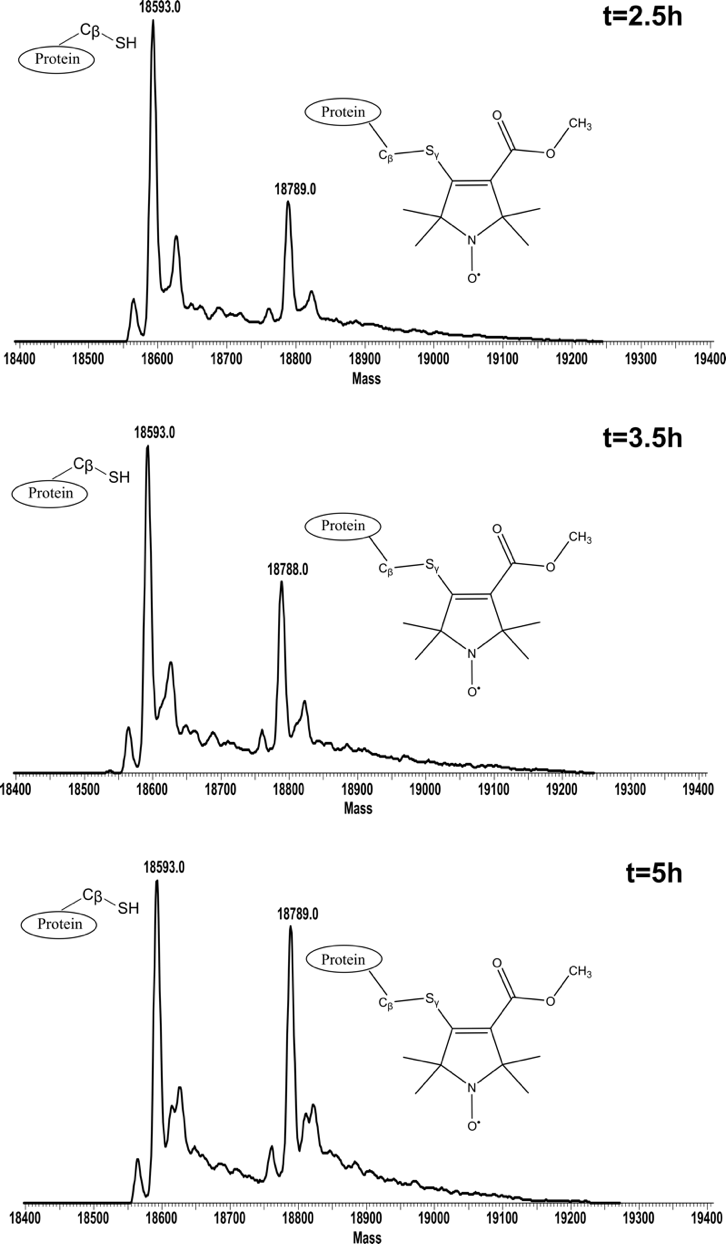


**Fig. S1** Deconvoluted ESI mass spectra of a reaction mixture of T4L mutant with a single solvent-exposed cysteine (72C) with HO-4072 after 2.5h, 3.5h, or 5 h.


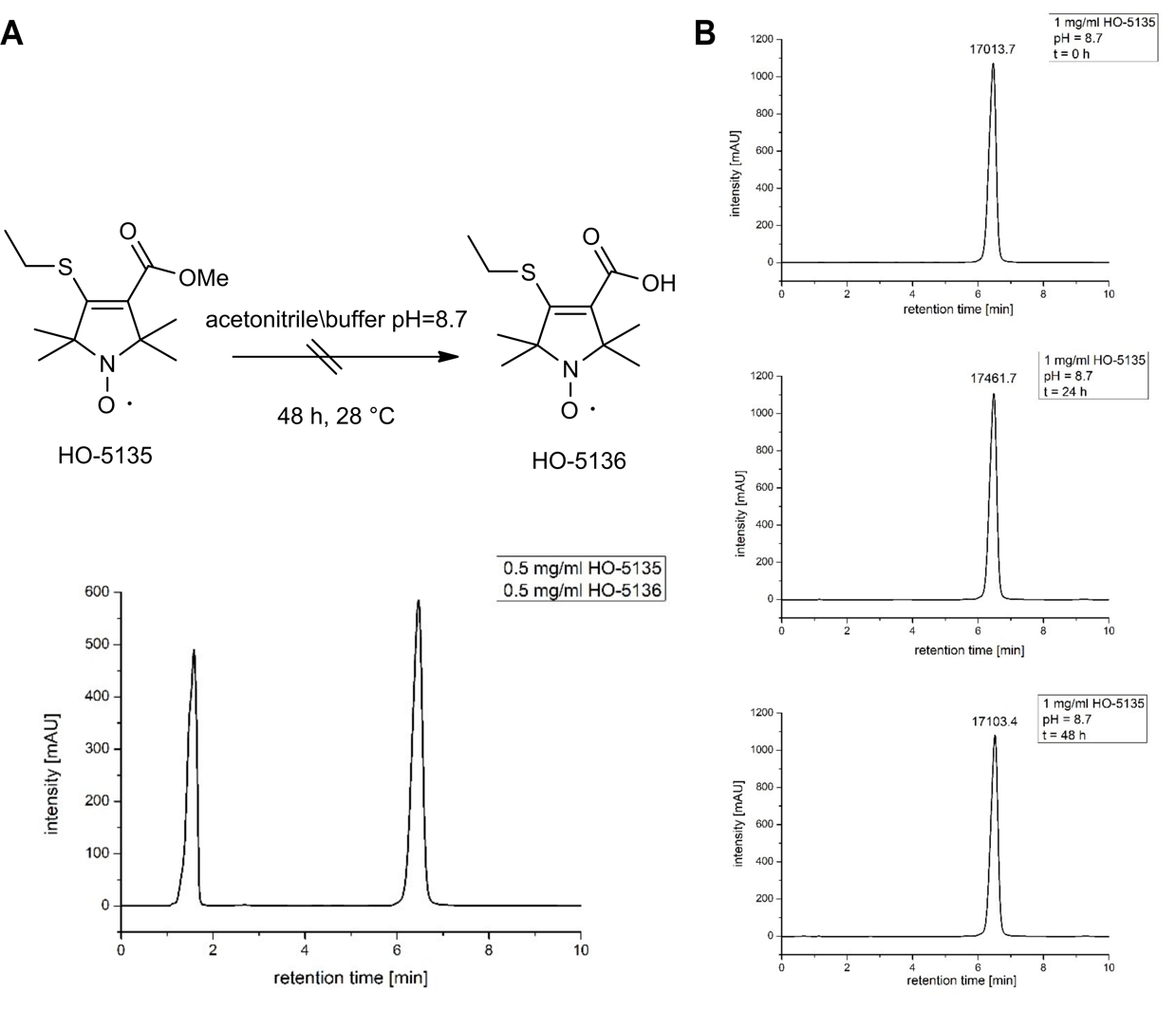


**Fig. S2** HPLC characterization results for ester hydrolysis of a thioether analogue of R9, HO-5135 **(A)** Reaction scheme and corresponding HPLC characteristic peaks. **(B)** The HPLC elution profiles monitoring unreacted HO-5135 as a function of time at pH 8.7, 28 ℃, over 48 hours.


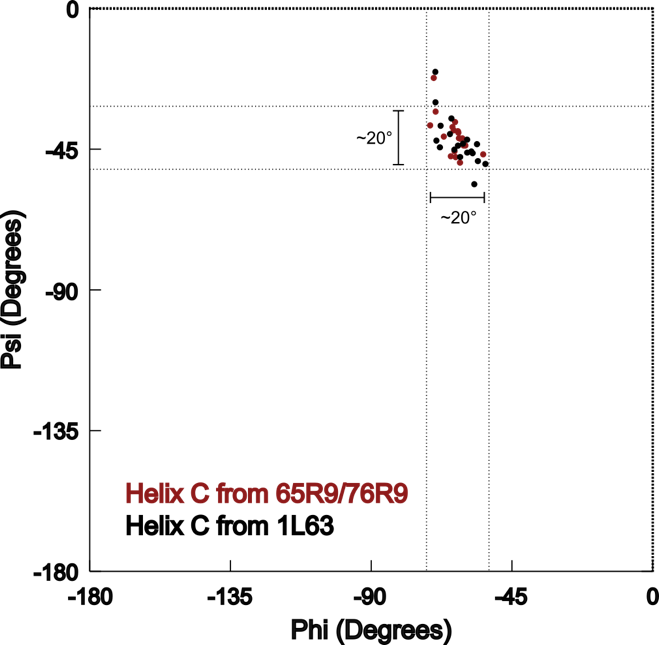


**Fig. S3** Ramachandran plot of the backbone dihedral angles for the T4L pseudo-wild type (PDB code 1L63) and 65R9/76R9 crystal structures, showing that R9 does not substantially perturb the local protein structure from the wild type.


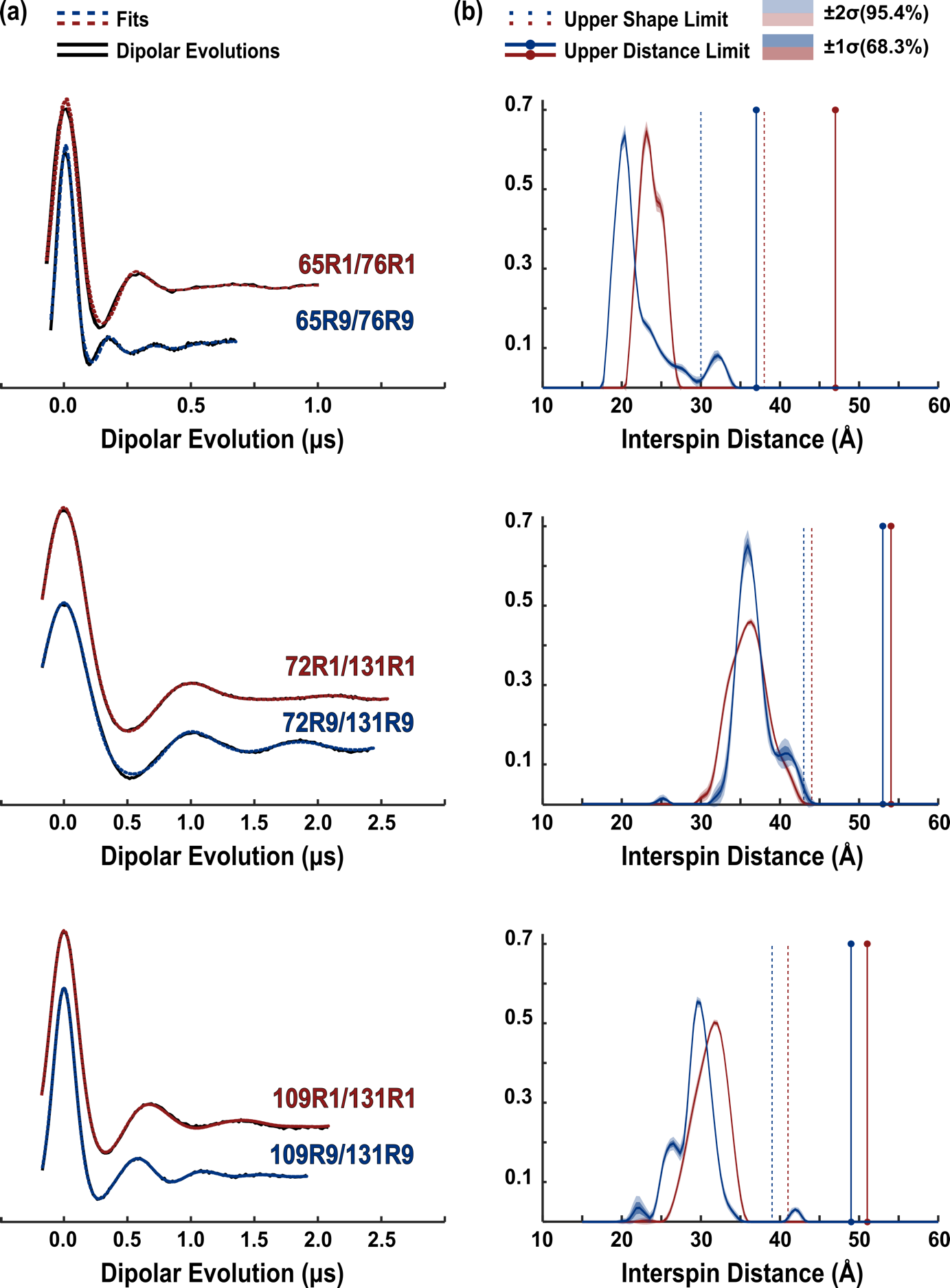


**Fig. S4** Model-free analysis and validations of interspin distance distributions measured by DEER **(a)** Background-subtracted dipolar evolutions of the indicated T4L mutants bearing either two R9 side chains (blue) or two R1 side chains (red). **(b)** Corresponding probability distance distributions (normalized) obtained from fits using model-free analysis and the confidence intervals (as mean distance ±1 or 2 standard deviation σ, accumulation 10000) of distance distributions generated by the program LongDistances. The upper limits for an accurate determination of the distances and the shape of distance distributions are also approximated and indicated in the figure as vertical lines located at the corresponding distances.


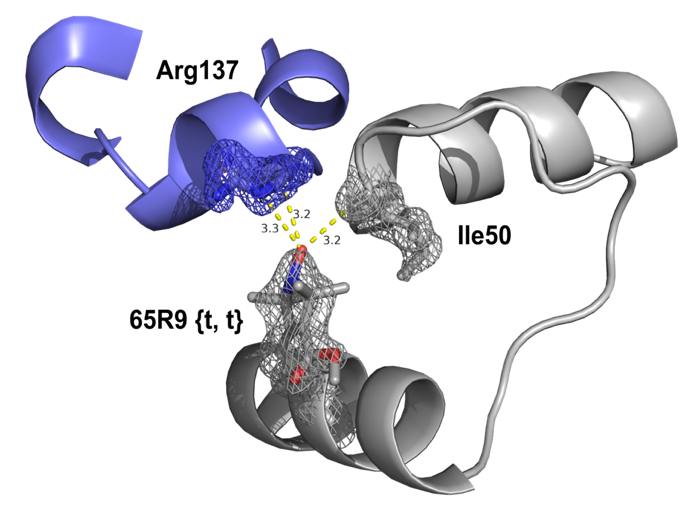


**Fig. S5** Interactions determined from the crystal structure between 65R9 {t, t} with Ile50 from the same protein molecule (grey), and Arg137 from a symmetry-related protein molecule in the crystal lattice (blue).


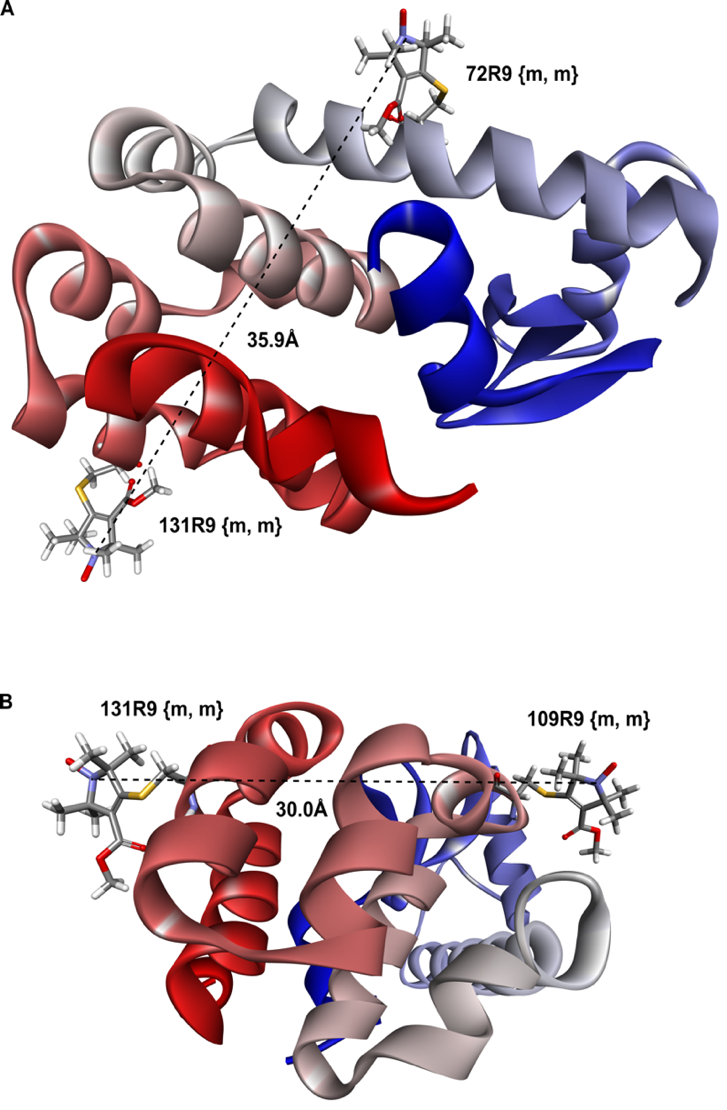


**Fig. S6** Models of T4L containing 72R9, 109R9, and 131R9 show the interspin distance corresponding to DEER measurements. **(A)** 72R9/131R9; **(B)** 109R9/131R9. The protein is colored as a transition from blue at its N-terminus to red at its C-terminus.

**References**

1. H. S. Mchaourab, M. A. Lietzow, K. Hideg, and W. L. Hubbell, Motion of Spin-Labeled Side Chains in T4 Lysozyme. Correlation with Protein Structure and Dynamics. Biochemistry. **35**, 7692–7704 (1996). https://doi.org/10.1021/bi960482k

2. M. R. Fleissner, M. D. Bridges, E. K. Brooks, D. Cascio, T. Kálai, K. Hideg, and W. L. Hubbell, Structure and dynamics of a conformationally constrained nitroxide side chain and applications in EPR spectroscopy. Proc. Natl. Acad. Sci. **108**, 16241–16246 (2011). https://doi.org/10.1073/pnas.1111420108

3. D. T. Warshaviak, V. V. Khramtsov, D. Cascio, C. Altenbach, and W. L. Hubbell, Structure and dynamics of an imidazoline nitroxide side chain with strongly hindered internal motion in proteins. J. Magn. Reson. **232**, 53–61 (2013). https://doi.org/10.1016/j.jmr.2013.04.013

4. M. Matsumura and B. W. Matthews, Control of Enzyme Activity by an Engineered Disulfide Bond. Science. **243**, 792–794 (1989). https://doi.org/10.1126/science.2916125

5. L. J. Perry and R. Wetzel, Unpaired cysteine-54 interferes with the ability of an engineered disulfide to stabilize T4 lysozyme. Biochemistry. **25**, 733–739 (1986). https://doi.org/10.1021/bi00351a034

6. M. Matsumura, W. J. Becktel, M. Levitt, and B. W. Matthews, Stabilization of phage T4 lysozyme by engineered disulfide bonds. Proc. Natl. Acad. Sci. **86**, 6562–6566 (1989). https://doi.org/10.1073/pnas.86.17.6562

7. C. J. López, M. R. Fleissner, Z. Guo, A. K. Kusnetzow, and W. L. Hubbell, Osmolyte perturbation reveals conformational equilibria in spin-labeled proteins. Protein Sci. **18**, 1637–1652 (2009). https://doi.org/10.1002/pro.180

8. C. J. López, M. R. Fleissner, E. K. Brooks, and W. L. Hubbell, Stationary-Phase EPR for Exploring Protein Structure, Conformation, and Dynamics in Spin-Labeled Proteins. Biochemistry. **53**, 7067–7075 (2014). https://doi.org/10.1021/bi5011128

9. E. G. Rozantsev and H. Ulrich, Syntheses of Some Stable Radicals and the Most Important Intermediates. In: edited by E. G. Rozantsev and H. Ulrich Free Nitroxyl Radicals. pp. 203–246. Springer US, Boston, MA (1970)

10. J. P. Whitelegge, J. le Coutre, J. C. Lee, C. K. Engel, G. G. Privé, K. F. Faull, and H. R. Kaback, Toward the bilayer proteome, electrospray ionization-mass spectrometry of large, intact transmembrane proteins. Proc. Natl. Acad. Sci. **96**, 10695–10698 (1999). https://doi.org/10.1073/pnas.96.19.10695

11. D. S. Cafiso and W. L. Hubbell, Estimation of transmembrane pH gradients from phase equilibriums of spin-labeled amines. Biochemistry. **17**, 3871–3877 (1978). https://doi.org/10.1021/bi00611a030

12. D. S. Cafiso and W. L. Hubbell, Estimation of transmembrane potentials from phase equilibriums of hydrophobic paramagnetic ions. Biochemistry. **17**, 187–195 (1978). https://doi.org/10.1021/bi00594a028

13. W. L. Hubbell, W. Froncisz, and J. S. Hyde, Continuous and stopped flow EPR spectrometer based on a loop gap resonator. Rev. Sci. Instrum. **58**, 1879–1886 (1987). https://doi.org/10.1063/1.1139536

14. J. S. Hyde and W. Froncisz, Loop gap resonators. Advanced EPR: Applications in Biology and Biochemistry. 277–306 (1989)

15. E. Meirovitch, A. Nayeem, and J. H. Freed, Analysis of protein-lipid interactions based on model simulations of electron spin resonance spectra. J. Phys. Chem. **88**, 3454–3465 (1984). https://doi.org/10.1021/j150660a018

16. D. J. Schneider and J. H. Freed, Calculating Slow Motional Magnetic Resonance Spectra. In: edited by L. J. Berliner and J. Reuben Spin Labeling: Theory and Applications. pp. 1–76. Springer US, Boston, MA (1989)

17. A. Polimeno and J. H. Freed, Slow Motional ESR in Complex Fluids: The Slowly Relaxing Local Structure Model of Solvent Cage Effects. J. Phys. Chem. **99**, 10995–11006 (1995). https://doi.org/10.1021/j100027a047

18. D. E. Budil, S. Lee, S. Saxena, and J. H. Freed, Nonlinear-Least-Squares Analysis of Slow-Motion EPR Spectra in One and Two Dimensions Using a Modified Levenberg–Marquardt Algorithm. J. Magn. Reson. **120**, 155–189 (1996). https://doi.org/10.1006/jmra.1996.0113

19. R. Owenius, M. Engström, M. Lindgren, and M. Huber, Influence of Solvent Polarity and Hydrogen Bonding on the EPR Parameters of a Nitroxide Spin Label Studied by 9-GHz and 95-GHz EPR Spectroscopy and DFT Calculations. J. Phys. Chem. A. **105**, 10967–10977 (2001). https://doi.org/10.1021/jp0116914

20. L. J. Berliner, editor , Spin labeling: theory and applications. Academic Press, New York (1976)

21. L. J. Berliner, editor , Spin labeling II: theory and applications. Academic Press, New York (1979)

22. D. Marsh, Spin-label electron paramagnetic resonance spectroscopy. CRC Press/Taylor & Francis Group, Boca Raton (2020)

23. D. D. Thomas, L. R. Dalton, and J. S. Hyde, Rotational diffusion studied by passage saturation transfer electron paramagnetic resonance. J. Chem. Phys. **65**, 3006–3024 (1976). https://doi.org/10.1063/1.433512

24. T. C. Squier and D. D. Thomas, Methodology for increased precision in saturation transfer electron paramagnetic resonance studies of rotational dynamics. Biophys. J. **49**, 921–935 (1986). https://doi.org/10.1016/S0006-3495(86)83720-1

25. D. D. Thomas, C. H. Wendt, W. Francisz, and J. S. Hyde, Saturation transfer EPR spectroscopy on spin-labeled muscle fibers using a loop-gap resonator. Biophys. J. **43**, 131–135 (1983). https://doi.org/10.1016/S0006-3495(83)84332-X

26. M. D. Bridges, Z. Yang, C. Altenbach, and W. L. Hubbell, Analysis of Saturation Recovery Amplitudes to Characterize Conformational Exchange in Spin-Labeled Proteins. Appl. Magn. Reson. **48**, 1315–1340 (2017). https://doi.org/10.1007/s00723-017-0936-3

27. G. Jeschke, DEER Distance Measurements on Proteins. Annu. Rev. Phys. Chem. **63**, 419–446 (2012). https://doi.org/10.1146/annurev-physchem-032511-143716

28. H. Russell, R. Cura, and J. E. Lovett, DEER Data Analysis Software: A Comparative Guide. Frontiers in Molecular Biosciences. **9**, (2022). https://doi.org/10.3389/fmolb.2022.915167

29. W. Kabsch, XDS. Acta. Cryst. D. **66**, 125–132 (2010). https://doi.org/10.1107/S0907444909047337

30. L. H. Weaver and B. W. Matthews, Structure of bacteriophage T4 lysozyme refined at 1.7 Å resolution. J. Mol. Biol. **193**, 189–199 (1987). https://doi.org/10.1016/0022-2836(87)90636-X

31. A. J. McCoy, R. W. Grosse-Kunstleve, P. D. Adams, M. D. Winn, L. C. Storoni, and R. J. Read, Phaser crystallographic software. J. Appl. Cryst. **40**, 658–674 (2007). https://doi.org/10.1107/S0021889807021206

32. P. D. Adams, R. W. Grosse-Kunstleve, L.-W. Hung, T. R. Ioerger, A. J. McCoy, N. W. Moriarty, R. J. Read, J. C. Sacchettini, N. K. Sauter, and T. C. Terwilliger, PHENIX: building new software for automated crystallographic structure determination. Acta. Cryst. D. **58**, 1948–1954 (2002). https://doi.org/10.1107/S0907444902016657

33. P. Emsley and K. Cowtan, Coot: model-building tools for molecular graphics. Acta. Cryst. D. **60**, 2126–2132 (2004). https://doi.org/10.1107/S0907444904019158
